# Hybrid Plasmonic Nanomaterials for Hydrogen Generation and Carbon Dioxide Reduction

**DOI:** 10.1021/acsenergylett.1c02241

**Published:** 2022-01-24

**Authors:** Simone Ezendam, Matias Herran, Lin Nan, Christoph Gruber, Yicui Kang, Franz Gröbmeyer, Rui Lin, Julian Gargiulo, Ana Sousa-Castillo, Emiliano Cortés

**Affiliations:** † Faculty of Physics, Ludwig-Maximilians-Universität, 80539 München, Germany

## Abstract

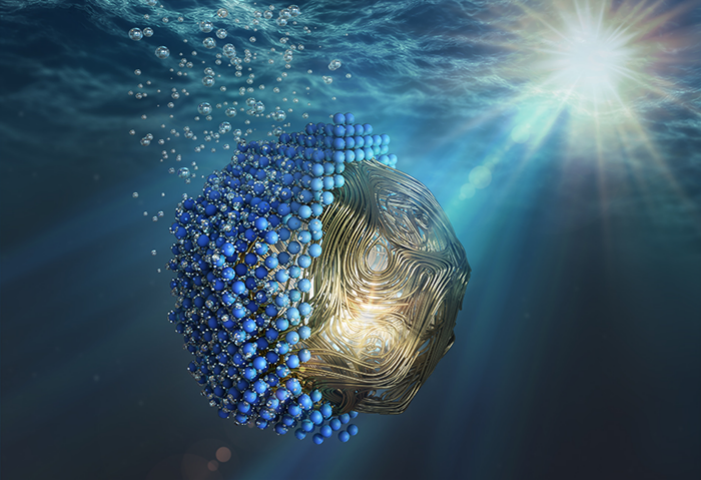

The successful development of artificial photosynthesis requires finding new materials able to efficiently harvest sunlight and catalyze hydrogen generation and carbon dioxide reduction reactions. Plasmonic nanoparticles are promising candidates for these tasks, due to their ability to confine solar energy into molecular regions. Here, we review recent developments in hybrid plasmonic photocatalysis, including the combination of plasmonic nanomaterials with catalytic metals, semiconductors, perovskites, 2D materials, metal–organic frameworks, and electrochemical cells. We perform a quantitative comparison of the demonstrated activity and selectivity of these materials for solar fuel generation in the liquid phase. In this way, we critically assess the state-of-the-art of hybrid plasmonic photocatalysts for solar fuel production, allowing its benchmarking against other existing heterogeneous catalysts. Our analysis allows the identification of the best performing plasmonic systems, useful to design a new generation of plasmonic catalysts.

Over the past two centuries, the rapid and continuing increase in energy consumption has led to a global energy and climate crisis. In the past few decades, almost 75% of human-caused carbon dioxide (CO_2_) emissions were a result of burning fossil fuels.^1^ Yet, to this date their share makes up more than 80% of the energy mix.^2^ It is evident that a transformation toward renewable energy sources is urgently needed. For millions of years, nature has used the sun as its primary energy source by photosynthesis during which sunlight is used to split water and produce energy-rich chemical compounds from CO_2_. Taking inspiration from this, artificial photosynthesis has become one of the most important research fields in the past decade. It consists mainly of two types of processes: one is water-splitting into hydrogen (H_2_) and oxygen (O_2_) or the H_2_ production from H_2_-containing compounds, and the other is the reduction of CO_2_ into fuels and chemicals, such as carbon monoxide, hydrocarbons, and oxygenates. These products are generally referred to as solar fuels.

In artificial photosynthesis, the first step is to harvest and concentrate the solar energy in molecular-sized regions. Plasmonic nanoparticles (PNPs) are promising materials to perform these tasks, due to their exceptional absorption and their ability to control light and heat at the nanoscale.^3−6^ Therefore, the inclusion of a plasmonic material into a multicomponent nanostructure can greatly enhance its photocatalytic performance compared to the individual components. In recent years, an increasing number of these so-called hybrid plasmonic nanostructures have been investigated for chemical reactions, giving rise to the field of plasmon-assisted catalysis.^7−12^

Despite being a young field, there are already several reviews,^13−15^ perspectives,^16−18^ and even a book^19^ covering different topics of the area. Some focus on the theoretical aspects and the working mechanisms of plasmonic photocatalysis.^20−22^ Others discuss reactions driven by a particular plasmon-induced phenomenon such as hot-carrier generation,^23^ photothermal effects^24^ or both.^25^ Furthermore, Devasia et al. focus on the possibility of controlling reaction pathways with plasmonic systems.^26^ Finally, some reviews focus on particular reactions, such as the applications of plasmonic catalysts to organic transformations^27^ or the CO_2_ reduction reaction (CO_2_RR).^28^ Among the reviews on plasmon-assisted catalysis there are few on hybrid systems, so far mostly limited to bimetallic or plasmonic metal–semiconductor systems.^29−33^

After around 10 years of exponential growth of the field, it is necessary to benchmark plasmonic materials for catalysis, not only among them but also against other existing heterogeneous catalysts for solar fuel production. This could give a real dimension of the improvements and achievements so far while showing the potential of this new type of heterogeneous (photo)catalysis in the quest of sustainable catalysis and clean energy production. Here, we will center on the considerable advances in the development of hybrid nanomaterials toward the production of solar fuels via plasmon excitation in the liquid phase. Many systems with great potential have been investigated for plasmonic catalysis in the gas phase.^9,11,25,34−40^ However, in this Review, we are limiting the examples to reactions under mild reaction conditions (i.e., heterogeneous catalysis in the liquid phase). In addition, we will narrow the scope of this Review to the reactions that are relevant for solar fuel production, namely H_2_ generation and CO_2_ reduction, but we will be as broad as possible regarding the evaluation of different types of novel hybrid systems, i.e., not only limited to metals and semiconductors but also including plasmonic hybrids with perovskites, 2D materials, and metal–organic frameworks for both pure photocatalysis and photoelectrocatalysis. With the increasing complexity and diversity of hybrid plasmonic nanostructures comes the necessity of a quantitative comparison among them. Here, we propose a series of standard metrics that should be reported for future plasmonic catalysts. Identifying the best performers is essential to design future plasmonic platforms for solar fuel production. That is the scope of this Review.

We first briefly elaborate on the motivation to employ plasmonic materials for photo(electro)catalysis and enumerate the enhanced properties that can be obtained in hybrid nanostructures. Second, we describe the reactions to produce solar fuels that are the focus of interest of this Review. These are the reactions toward CO_2_ reduction and H_2_ generation. Third, we present the performance metrics that will be used to compare different materials/systems. In the main section, recent developments in each of the hybrid systems are reviewed. Last, in the Discussion section, the systems are summarized and compared for each reaction in a concluding meta-analysis.

## Properties of Hybrid Plasmonic Nanomaterials

PNPs interact strongly with light through the excitation of localized surface plasmon resonances (LSPRs), which correspond to the coherent oscillations of their free electrons coupled to the electromagnetic field.^3^ Plasmonic materials are typically metals with negative real permittivity (i.e., with high concentration of free electrons) such as silver, gold, copper, etc. The plasmon resonances are highly tunable, and their frequency can cover the ultraviolet (UV), visible, and near-infrared (NIR) regions of the electromagnetic spectrum (i.e., the totality of solar emission).^5^ When resonantly illuminated, the absorption cross sections of PNPs reach tens of times their geometrical size, making them efficient harvesters of light. In addition, PNPs can act as solar light antennas. They can concentrate light in small volumes of typically a few nm^3^ near their surface, where the intensity can reach values as high as 10^5^ the solar one.^4^ That volume is on the order of molecular sizes, transforming the PNPs into nanoreactors within molecular scales.

There is a variety of plasmon-driven processes that can affect the activity and selectivity of target molecules.^20,41^ Plasmon resonances are of mixed optoelectronic nature, and as such, the energy from incoming light is partly stored in the kinetic energy of charge carriers and in the electromagnetic field. These resonances can decay radiatively or transfer their energy to a charge carrier. This highly energetic charge carrier then relaxes, during which secondary and lower energy charge carriers are excited, and ultimately heat is released to the surrounding of the PNPs. This means that a resonantly illuminated PNP can supply a broad range of energy quanta, from highly energetic charge carriers with the energy of the incoming photons (∼2–3 eV) to thermal phonons (∼0.025 eV). A nearby molecule or material can use these energy quanta to perform a chemical transformation in thermal or non-thermal ways. The versatility of plasmon-driven processes offers the opportunity to obtain alternative reaction pathways toward a desired but kinetically or thermodynamically unfavorable product. For example, they can operate in a particular intermediate step of a reaction, altering the pathway toward a different product.^42,43^

**Figure 1:**
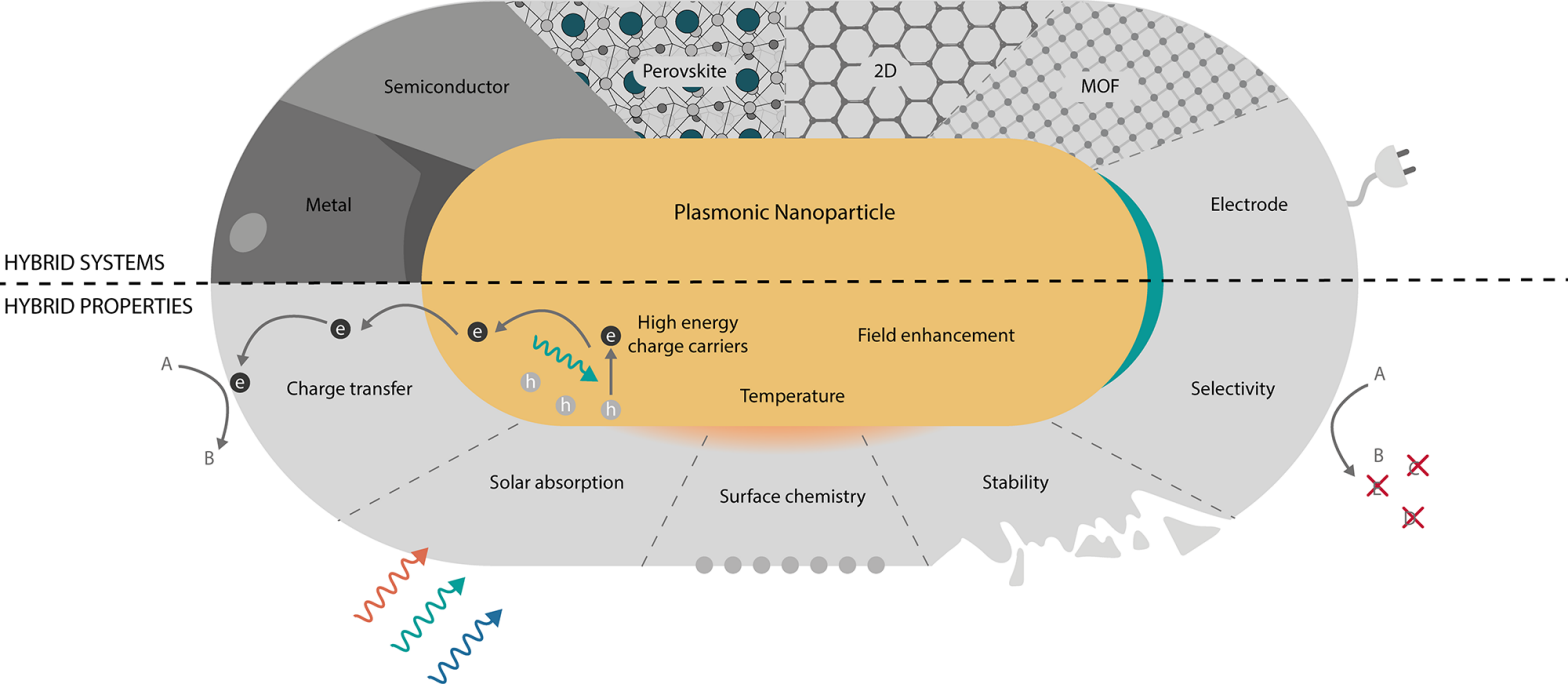
Hybrid plasmonic photocatalysts. (Top) Overview of the hybrid plasmonic systems that are the topic of this Review. From left to right: metal, semiconductor, perovskite, 2D, MOF, and electrochemistry. (Bottom) Desirable properties of an ideal photocatalyst. The plasmonic component can harvest light and transfer the energy through charge carriers, heat, and electromagnetic fields. Non-plasmonic materials can add or enhance catalytic properties. The hybrid system can show enhanced stability, selectivity, or activity through various mechanisms, e.g., effective charge transfer, surface chemistry, or enhanced absorption.

However, the catalytic activity of typical plasmonic metals (Au, Ag, Cu, etc.) for solar fuel production is comparatively low.^44,45^ In recent years, plasmonic components have been interfaced with other nanomaterials, searching for superior hybrid plasmonic nanocatalysts. These hybrid plasmonic catalysts are summarized in the top half of Figure 1. The promising properties of PNPs are combined with additional enhanced photocatalytic properties from the second material that are in many cases not present in the single-component counterparts. The bottom half of Figure 1 schematizes some of the properties that are desirable (and potentially obtainable) in a hybrid plasmonic catalyst. While the plasmonic component harvests light and supplies energy through charge carriers, heat, or electromagnetic fields, a second (non-plasmonic) component can, for example, improve the interaction with target molecules, enhance the stability of the system or enlarge the lifetime of excited carriers. In the following, we present the particular opportunities that each material/interface opens for plasmonic catalysis.

### Plasmonic Metal–Metal

The catalytic activity of plasmonic metals can be improved by combining plasmonic metals with common catalytically active metals such as palladium, platinum, rhodium, etc. The inclusion of such metals can help lower the activation energy of a reaction step or enhance the adsorption/desorption dynamics of reactants/products on the surface of the nanocatalyst. In addition, combining the two metals modifies the electronic energy bands close to their interface, potentially facilitating charge flow, reducing the chemical potential of the charge carriers, and enhancing their extraction.^46^ In bimetallic systems, the decay of plasmon resonances gets altered, favoring non-radiative (excitation of carriers, heat generation) versus radiative channels (scattering).^47−49^ Interestingly, the spatial distribution of absorption can be shifted toward the catalytic metal generating carriers exactly where they are more reactive.^38,50,51^

### Plasmonic Metal–Semiconductor

The lifetime of photoexcited charge carriers in plasmonic metals is typically less than 1 ps.^52,53^ This time is usually too short for the carriers to act on an adsorbate, a detrimental fact for the overall reactivity of the system. Longer charge carrier lifetimes can be attained by interfacing the metal with a semiconductor. The decay of the plasmon can generate charge carriers in the semiconductor through three main mechanisms. First, photoexcited electrons in the metal with sufficient energy can be transferred across the Schottky barrier^54^ to the conduction band of the semiconductor, in a process called indirect electron transfer.^24,55^ Second, a plasmon can decay by directly exciting an electron into the conduction band of the semiconductor and a hole in the metal, in a process called plasmon-induced charge-transfer transition^55^ or direct electron transfer.^13,56^ The third mechanism is a plasmon-induced resonant energy transfer,^57,58^ where an electron–hole pair is excited in the semiconductor. The presence of a Schottky barrier at a metal/semiconductor interface^54^ traps electrons and prevents or delays them from coming back to the metal.^55^ The lifetime, just like the indirect electron injection across the barrier, depends on the potential difference and a trade-off has to be made between the harvesting and recombination of carriers.^59,60^ Furthermore, structural defects and/or vacancies in the semiconductor can also act as electron traps, enhancing their lifetime, and are usually the most reactive sites.^61^ These processes allow electrons to become available in the semiconductor to drive reduction reactions while holes remain in the metal for oxidation reactions.^13^ Inversely, there have also been demonstrations of semiconductors harvesting the holes.^40,62^ Without the photoexcited electrons in the plasmonic metal, wide bandgap semiconductors like titanium dioxide (TiO_2_) are photocatalytically active only in the UV. The energy transfer mechanisms from the plasmonic material to the semiconductor extend their operational wavelength range, accommodating a larger fraction of the solar spectrum and potentially increasing the solar-to-fuel conversion efficiency.^63,64^

### Plasmonic Metal–Perovskite

Perovskites are a versatile subclass of semiconductors with attractive optoelectronic and catalytic properties. They are characterized by their ABX_3_ crystal structure, where A and B represent cations and X an anion (see Figure 4a, below). As semiconductors, the physical and chemical properties of perovskites leading to a catalytic enhancement are generally as described above. This is especially true for ABO_3_-type perovskite oxides, which are cost efficient and can accommodate a wide range of substituting and doping elements to modulate their electronic and catalytic properties but are generally photocatalytically active only under UV irradiation owing to their wide bandgap. Their combination with plasmonic materials allows the absorption of visible light, making irradiation of wavelengths with photon energies below the bandgap of the perovskite accessible.^65−68^ Both charge or energy transfer from the plasmonic material to the perovskite can occur.^69,70^ The perovskite oxides are in many ways very similar to semiconductors included in the general semiconductor section; However, the perovskite category is included in this Review due to a second type of perovskite, namely ABX_3_-type perovskite halides (X = F, Cl, Br, I). They have a tunable absorption across the entire visible spectrum^71^ as well as long carrier lifetimes.^72^ In addition to the benefits discussed above, by combining perovskite halides and plasmonic materials, the characteristic absorption spectra of both components can be matched such that the strong near-field enhancement of the PNP can significantly enhance the absorption of light by the halide perovskite, potentially boosting their catalytic performance.

### Plasmonic Metal–2D

A 2D material is the co-catalyst with the largest possible available surface. Of particular interest are materials with outstanding electrical conductivity and mobility, such as carbon-based 2D and transitional metal-based 2D materials. The photogenerated electrons in the PNPs can transfer to the 2D material, where they benefit from a larger lifetime fostering substrate reactivity.^73^ Furthermore, electrons can accumulate in the 2D material or promote the formation of charged excitons (with more than one electron coupled to a hole).^74,75^ This accumulation facilitates reactions that require multiple electrons. Finally, the presence of defects, dopants, or oxygenated functional groups in the 2D material can act as catalytic sites for specific reactions.^76^

### Plasmonic Metal–MOFs

Metal–organic frameworks (MOFs) are ordered periodic structures formed by nodes of metal ions linked by organic ligands.^77−79^ Plasmonic materials can gain interesting properties relevant to catalysis when combined with this versatile class of porous materials.^80−84^ First, MOFs can act as substrates, providing support and increasing stability.^85^ Second, they are highly versatile in composition and pore size, allowing structural tailoring according to the application. For example, their organic linker can be adjusted to catalyze a specific reaction or co-reaction.^86^ This property makes them promising systems to achieve high selectivity of product formation. Third, they can be structurally designed so that their catalytic centers lie in the near-field enhancement region of the plasmonic component, enhancing their absorption.^85^ Fourth, their high porosity can allow an extraordinarily large surface area for a reaction to occur^87^ or the preconcentration of reactants next to the PNPs.^88^ Finally, MOFs can also act as a charge carrier mediator to enhance the electron–hole pair separation efficiency.^89^

### Plasmon-Assisted Electrochemistry

Electrocatalysis, like photocatalysis, has been studied extensively in recent years.^90^ While promising, electrochemical reactions often require high overpotentials. Plasmonic materials can enhance the performance of electrocatalytic systems by utilizing the solar energy. This can result in lower electrical energy consumption and higher product formation, which manifest as a reduced overpotential or increased current density.^91−93^ The introduction of a plasmonic material can also modify the chemical landscape, which can affect selectivity by triggering or enhancing otherwise thermodynamically and/or kinetically hindered reactions.^94^

## Plasmon-Assisted Reactions toward Solar Fuel Generation

In this Review, we discuss hybrid photocatalysts with at least one plasmonic component that is resonantly illuminated. This excludes, for example, systems where the presence of a plasmonic metal can have a positive impact on the catalytic activity of the system but is either not illuminated or illuminated in a region with negligible absorption.^95,96^ It should be noted that we consider both photocatalytic processes (typically thermodynamically favorable, Δ*G* < 0, with *G* the Gibbs free energy) and photosynthetic processes (thermodynamically unfavorable, Δ*G* > 0) as plasmon-assisted reactions. For simplicity, they will from here on be referred to as photocatalytic. Among the large list of recently demonstrated plasmon-assisted reactions, we focus on a few particularly relevant reactions toward the generation of solar fuels: H_2_ generation reactions and CO_2_RRs, as described below and summarized in Table 1.

**Table 1:** Reactions toward Solar Fuel Production

reaction	fuel
**H_2_ Production**	
NH_3_BH_3_ + 3H_2_O → NH_4_B(OH) + 3H_2_*(ammonia borane hydrolytic dehydrogenation)*	hydrogen
NaBH_4_ + 4H_2_O → NaB(OH)^4^ + 4H_2_*(sodium borohydride hydrolytic dehydrogenation)*	hydrogen
HCOOH → CO_2_ + H_2_*(formic acid dehydrogenation)*	hydrogen
2H_2_O + 4e^–^ → 2O_2_ + H_2_ (*water-splitting*)	hydrogen
	
**CO_2_ Reduction**	
CO_2_ + 2H^+^ + 2e^–^ → CO + H_2_O	carbon monoxide
CO_2_ + 2H^+^ + 2e^–^ → HCOOH	formic acid
CO_2_ + 4H^+^ + 4e^–^ → HCHO + H_2_O	formaldehyde
CO_2_ + 6H^+^ + 6e^–^ → CH_3_OH + H_2_O	methanol
CO_2_ + 8H^+^ + 8e^–^ → CH_4_ + 2H_2_O	methane
2CO_2_ + 12H^+^ + 12e^–^ → C_2_H_5_OH + 3H_2_O	ethanol

### Hydrogen Generation

Because hydrogen can be burned with no greenhouse emissions, it is a promising candidate to replace fossil fuels. Hydrogen has a high gravimetric energy density of 33.3 kW h kg^–1^, which is almost 3 times higher than that of gasoline (12.4 kW h kg^–1^).^97,98^ It is stable and can be liquefied, stored, or transported through pipelines. However, the majority of hydrogen is still produced from fossil fuels, resulting in excessive carbon emissions.^99^ For hydrogen to become a sustainable fuel, it has to be produced from renewable energy sources.

Photocatalytic hydrogen sources can be categorized as follows: (1) *Water*. Solar hydrogen production via water-splitting is especially attractive, as both water and sunlight are abundant. Materials for photocatalytic water-splitting are either investigated toward their performance of full water-splitting, where H_2_ and O_2_ evolution should occur in a stoichiometric 2:1 ratio, or by using a sacrificial hole acceptor, for only the hydrogen evolution reaction (HER). This simplifies research and prevents charge recombination. However, this sacrificial agent should also be cheap and abundant, as otherwise this could become a new bottleneck for the reaction to be scaled up. (2) *Other hydrogen-containing compounds.* While hydrogen has a high energy content per mass, its energy content per volume is poor. This makes storing enough hydrogen, e.g., on-board of means of transport, difficult. A possible solution is the chemical storage in compounds with a higher storage density. This has resulted in compounds with a high hydrogen content like formic acid (HCOOH),^100^ ammonia borane (NH_3_BH_3_),^101^ or sodium borohydride (NaBH_4_)^102^ gaining attention, especially for mobile and portable applications.

### Carbon Dioxide Reduction

Carbon dioxide (CO_2_) is one of the main greenhouse gases contributing to climate change.^103^ CO_2_ conversion can have the two-fold benefit of decreasing its atmospheric concentration while also producing fuels. Compared to hydrogen production, the reactions for CO_2_ conversion are much more complex, as a variety of reaction pathways coexist. Possible products include carbon monoxide (CO), formic acid (HCOOH), methane (CH_4_), methanol (CH_3_OH), ethane (C_2_H_6_), propane (C_3_H_8_), and ethanol (CH_3_CH_2_OH), among others. These products require a different number of charge transfers, as shown in Table 1, and contain different numbers of carbon. Products with two or more carbon atoms, so-called C2+ products, are desirable but difficult to produce. Several steps can be involved, leading to the possibility of branching reaction pathways and various products being produced simultaneously. Therefore, a suitable large-scale photocatalyst for CO_2_ reduction should not only present a high efficiency but also a high selectivity to one product. In this Review, we will quantitatively evaluate the prospect of using hybrid plasmonics toward these goals.

## Performance Metrics

The main challenge when comparing different hybrid nanocatalysts is to find quantitative and relevant performance metrics. Different aspects of a catalyst performance may be evaluated, such as its activity, selectivity, stability, or temperature of operation. However, it is not straightforward to determine metrics depending only on the nanocatalyst’s intrinsic properties because its performance may be strongly altered by reaction conditions such as the illumination characteristics or reactant concentration. The typically reported performance metrics used in the field are discussed below. Table 2 summarizes the metrics that will be extracted from the original publications and written in tables for comparison.

**Table 2:** Performance Metrics for Hybrid Plasmonic Catalysts That Are Used in This Review

	photocatalysts	photoelectrocatalysts
activity	formation rate [μmol g^–1^ h^–1^]	overpotential [mV] @ current density of 10 mA cm^–2^
	apparent quantum efficiency (AQE) [%]	onset potential [mV]
		Tafel slope [mV dec^–1^]

selectivity	selectivity [%]	Faradaic efficiency (FE) [%]

stability	stability [h]	stability [h]
	duration [h]	duration [h]

temperature	reactor temp [°C]	reactor temp [°C]

light enhancement,	activity enhancement factor (LE,PE)	shift in operation point [ΔmV or ΔmV dec^–1^]
plasmonic enhancement		

*Activity* is quantified by the formation rate of a product (amount of generated product per time per mass of hybrid catalyst, eq 1). It must be noted that this metric assumes a linear relation of product formation with respect to time and catalyst concentration, which is often not the case.^104^
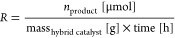
1In this Review, we will use the formation rate (eq 1) as the main metric for activity, as it is most commonly reported. If it is not possible to extract the formation rate, the turnover frequency (TOF) or turnover number (TON = TOF × time) is reported in the tables. Across the literature, the TOF is used with conflicting definitions. In hybrid nanomaterials where the reaction occurs in only one of the components, the number of catalytic sites might be used instead of the total mass of the catalyst. This value is rarely reported as it is difficult to determine because the information on the number of active catalytic sites is not always available or might fluctuate in time. Instead, TOF is more commonly referred to as the mole of product molecules per mole of catalyst per time (eq 2).
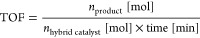
2The key role of illumination in plasmon-assisted catalysis is not reflected in the activity metrics discussed so far. Therefore, the quantum efficiency (QE) should be used as a complementary metric (eq 3).^105^ It is defined as the number of reacted electrons per absorbed photon and determined for a specific wavelength. Whenever a quantum efficiency is reported, it is usually the apparent quantum efficiency (AQE), defined as the number of reacted electrons per incident photon (eq 4). This is the case because the number of absorbed photons is often difficult to determine. The AQE is a lower bound for the QE and will be used in this Review whenever it is reported.
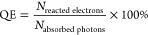
3
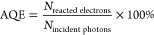
4In photoelectrocatalysis, the metrics reported are different from the ones for photocatalysis. A metric for the activity is the overpotential (mV) at a given current density (mA cm^–2^) (or vice versa). The onset potential (mV), the potential at which the reaction starts to occur, describes the reaction energy barrier. Finally, the Tafel slope (mV dec^–1^), obtained by plotting the overpotential as a function of the logarithm of the current density, is used to estimate the reaction kinetics of the system.

*Selectivity* is a major concern for CO_2_ conversion. For photocatalytic systems, it is generally reported as the ratio between the formation rate of the major product and all products in percent (eq 5).
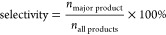
5For electrocatalytic systems, the Faradaic efficiency (FE) is more commonly reported. It describes the proportion of reacted electrons facilitating the formation of a certain product. The *stability* of the catalyst is referred to as the longest investigated time under operando conditions in which its activity did not significantly decrease. Whenever the stability was not examined, the experiment duration is reported in the table instead.

*Temperature* is an important parameter that modulates the rate of reactions through the Arrhenius law. Illuminated plasmonic systems offer the opportunity of driving reactions at mild conditions, without the need for high external temperatures. Here, whenever possible, the operating temperature of the reactor will be used as a metric. It must be noted that this value might strongly differ from the surface temperature of the nanocatalysts.^106^

The *light enhancement* (LE) aims to quantify the contribution of light to a given reaction. When doing this, one should also compensate for the temperature increase upon illumination;^107^ however, this is not frequently done. Here, it will therefore simply be estimated as the ratio between the activity (i.e., formation rate or TOF) of a nanocatalyst in the presence and in the absence of light.

Similarly, the *plasmonic enhancement* (PE) is the activity ratio of the illuminated system in the presence and absence of the plasmonic component. In photoelectrocatalysis, the enhancements are evaluated as a shift in the overpotential at a current density of 10 mA cm^–2^ of the reactions.

In the following, we will review the latest demonstrations of hybrid plasmonic nanomaterials for solar fuel production.

## Plasmonic Metal–Metal

**Figure 2:**
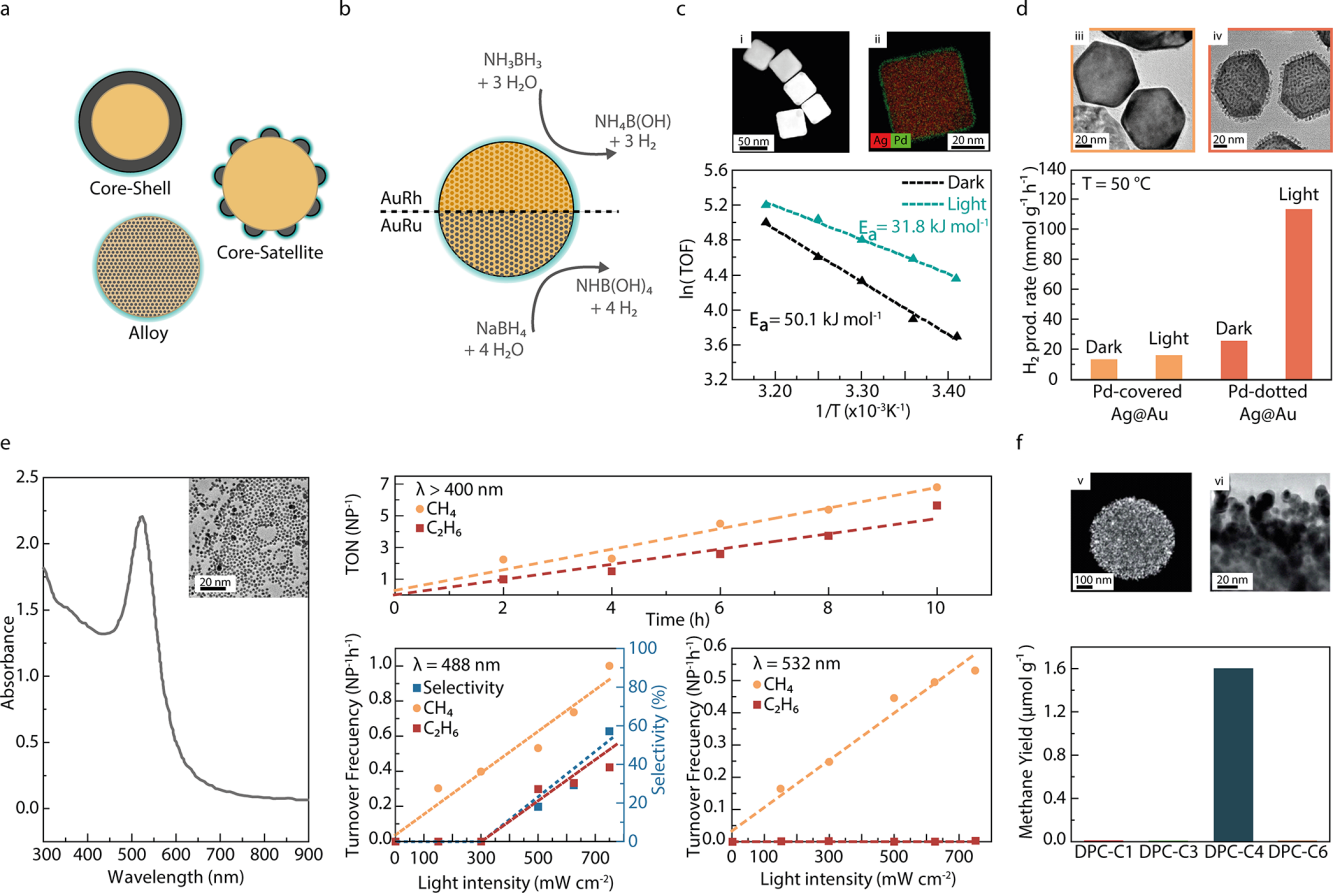
Plasmonic metal–metal hybrids. (a) Illustration of bimetallic hybrids with different configurations. (b) Reaction-catalyst pairs. Au_1_Rh_1_ performed better toward NH_3_BH_3_ dehydrogenation, whereas Au_1_Ru_1_ performed better toward NaBH_4_ dehydrogenation. (c) STEM-HAADF (i) and EDS (ii) images of Ag nanocubes (red) coated with an ultrathin Pd layer (green), employed on NH_3_BH_3_ dehydrogenation. Arrhenius plot in both dark and light. (d) TEM image of both Pd-covered (iii) and Pd-dotted (iv) Ag@Au nanohexagons and their performances toward FA dehydrogenation. (e) Extinction spectrum of ∼12 nm AuNPs used as catalyst in CO_2_RR. Top: cumulative number of product molecules generated per AuNP (TON) as a function of time under visible illumination (λ > 400 nm). Bottom: performance toward CO_2_RR under blue (λ = 488 nm) and green (λ = 532 nm) illumination as a function of power. (f) Top: STEM-HAADF (v) and TEM (vi) of Au colloidosomes utilized for CO_2_ reduction. Bottom: CH_4_ generation out of CO_2_ reduction for Au colloidosomes at different growth steps of AuNPs, varying its size and interparticle distance. (b) Adapted with permission from ref. (108). Copyright 2020 American Chemical Society. (c) Adapted with permission from ref. (109). Copyright 2020 American Chemical Society. (d) Adapted with permission from ref. (110) Copyright 2020 Elsevier. (e) Adapted with permission from ref. (111). Copyright 2018 American Chemical Society. (f) Adapted with permission from ref. (112). Copyright 2019 Royal Society of Chemistry.

In recent years, several configurations of hybrid plasmonic bimetallic nanoparticles ranging from alloys, core–shell, partially coated structures to self-assembled catalysts, have been employed to boost the H_2_ production and CO_2_ reduction through plasmon excitation (illustration in Figure 2a). A summary of these publications can be found in Table 3 and Table 4, including the performance metrics defined in the introduction, as well as their respective experimental conditions. In the following, we will describe selected examples on these topics.

### Plasmonic Metal–Metal Hybrids for H_2_ Generation

Toward more efficient photocatalytic H_2_ generation, plasmonic materials can benefit from the addition of late transition metals. Engineering the materials to exploit both light-harvesting and catalytic properties is one of the utmost challenges for these so-called plasmonic bimetallic systems. Next, we outline recent relevant reports in which different configurations were investigated for H_2_ production.

An important study on alloys, carried out by Kang et al., showed that the efficiency of the Au-based alloyed catalyst was dependent on both the chemical composition of the hybrid and the hydrogen source.^108^ Specifically, in their article Au was alloyed with different metals (Rh, Ru, Pt, Pd, Co, and Ni), and the resulting catalysts were tested toward the hydrolytic dehydrogenation of both ammonia borane (NH_3_BH_3_) and sodium borohydride (NaBH_4_). The best performers for both reactions are illustrated in Figure 2b. For the photocatalytic hydrolysis of NH_3_BH_3_, Au_1_Rh_1_, 1:1 atomic ratio alloys, showed the highest H_2_ production (TOF = 240 min^–1^), followed by its Pt-analogue (Au_1_Pt_1_, TOF = 200 min^–1^). For both systems, the influence of light was evidenced by the almost 3-fold increase compared to dark conditions. When compared to their respective monometallic counterparts, Rh and Pt, the hybrids showed a plasmonic H_2_ production enhancement of 2.4 and 1.8, respectively, indicating the benefits of such systems. However, for NaBH_4_ dehydrogenation, Au_1_Ru_1_ performed better than Au_1_Rh_1_, Au_1_Pt_1_, and the other alloys, achieving a production of TOF = 178 min^–1^. In addition to its superior H_2_ production, the Au_1_Ru_1_ hybrid was the catalyst that maximized the use of the incoming light. In comparison to the experiments in dark, the activity increased by a factor of 3.8 under illumination. Again, compared to the monometallic RuNPs, the inclusion of the plasmonic Au led to an improvement of 2.2-fold. This improved H_2_ production was a result of hot carrier injection from Au into the catalytically active Ru atoms, weakening the B–H bonds in the borohydride.

Unlike alloys, in core–shell structures, the adsorbates can only interact with the outermost metal, which typically is the catalytic metal. This introduces an important difference between the systems. Instead of isolated active centers, the entire surface of the core–shell structure becomes active for catalytic processes. Xu et al. fabricated a hybrid plasmonic photocatalyst by coating an Ag nanocube with a thin layer of Pd (Ag@Pd NC).^109^ Panels i and ii of Figure 2c illustrate the well-defined spatial arrangement of the metals, showing that Pd was placed only at the surface, encapsulating the Ag optical antenna. The Ag@Pd NCs were tested toward the hydrolytic dehydrogenation of NH_3_BH_3_, and changes in the activation barrier under visible light illumination were studied. The Ag@Pd NCs showed a H_2_ production rate of 102 × 10^6^ μmol g^–1^ h^–1^ at 40 °C, which is one of the highest rates in Table 3. This production under illumination was 1.2 times larger compared to dark conditions. In spite of this outstanding production, the most interesting finding is that the activation energy (*E*_a_) was lowered from 50 kJ mol^–1^ to 31.8 kJ mol^–1^ under simulated sunlight illumination, as shown at the bottom of Figure 2b. The authors attributed the lowered *E*_a_ barrier to the fact that excited electrons on Ag accumulated within the thin Pd shell. Those excited carriers would then populate the lowest unoccupied molecular orbital (LUMO) of the Pd–NH_3_BH_3_ complex at the interface, thus facilitating the H_2_ production.

**Table 3:** Plasmonic Metal–Metal Hybrid Systems for H_2_ Generation

photocatalyst	light source	reaction	temp (°C)	formation rate (μmol g^–1^ h^–1^)	stability (h)	AQE (%)	PE	LE	ref
**PNP–Metal for H**_**2**_									
Au_1_Rh_1_	7 W visible LED strips	NH_3_BH_3_ dehydrogenation	25	240 TOF (min^–1^)	>0.09^a^	–	2.4	2.8	(108)
Au_1_Pt_1_	7 W visible LED strips	NH_3_BH_3_ dehydrogenation	25	200 TOF (min^–1^)	>0.09^a^	–	1.8	2.7	(108)
Au_1_Ru_1_	7 W visible LED strips	NaBH_4_ dehydrogenation	25	178 TOF (min^–1^)	>0.42	–	2.2	3.8	(108)
Ag@Pd nanocubes	Xe lamp (100 mW cm^–2^)	NH_3_BH_3_ dehydrogenation	40	102 × 10^6^ c	>10	–	–	1.2	(109)
Zn_1.3_Cu_98.7_	solar simulator (788 mW cm^–2^)	H_2_O reduction (MeOH)	–	328 × 10^3^	>4	7 (@570 nm)	39	1.2	(113)
Pd-tipped Au nanorods^e^	300 W Xe lamp (λ > 400 nm)	FA dehydrogenation	45	133 × 10^3^	–	–	2.5	–	(114)
Pd-dotted Ag@Au hexagonal NPs	300 W Xe lamp (λ > 420 nm)	FA dehydrogenation	50	113 × 10^3^ c	>8	–	–	4.5	(110)
Pd-tipped Au nanorods	300 W Xe lamp (λ > 460 nm)	FA dehydrogenation	40	30 × 10^3^	–	–	–	3	(115)
Pd-covered Ag@Au hexagonal NPs	300 W Xe lamp (λ > 420 nm)	FA dehydrogenation	50	15 × 10^3^ c	>8	–	–	1.15	(110)
Au@Pd nanodog-bones	300 W Xe lamp	FA dehydrogenation	15	1050	>4^a^	–	b	d	(116)
Pt-edged Au nanoprisms	300 W Xe lamp (λ > 420 nm)	H_2_O reduction (MeOH)	–	0.167 μmol h^–1^	>6^a^	–	5	–	(117)
Pt-tipped Au nanoprisms	300 W Xe lamp (λ > 420 nm)	H_2_O reduction (MeOH)	–	0.031 μmol h^–1^	>6^a^	–	6.5	–	(117)

a Experiment duration.

b Negligible product detected without plasmonic material.

c Normalized only by catalytic metal.

d Negligible product detected without light.

e Supported by magnetic field.

By coating a plasmonic antenna with a catalytic material, the optical properties of the overall system are damped. Instead, partially coating the plasmonic components allows us to preserve the plasmon and still exploit the interface between both. Tong et al.^110^ compared the performance of hybrid systems in which the catalytic metal was either homogeneously or heterogeneously grown on a plasmonic antenna. Both Pd-covered and Pd-dotted Ag@Au hexagonal nanoparticles were fabricated and tested toward the generation of H_2_ out of formic acid (HCOOH) (Figure 2d). The Pd-covered Ag@Au showed a H_2_ production of 15 × 10^3^ μmol g^–1^ h^–1^ when illuminated with visible light at 50 °C with a light enhancement factor of 1.15. However, when the Pd was shaped into dots instead of a continuous shell, the performance was further improved up to 113 × 10^3^ μmol g^–1^ h^–1^. The control of the morphology of the active metal not only enabled a boost in H_2_ generation, but also resulted in a larger light enhancement. Indeed, the improvement of 4.5-fold in its activity under visible light illumination is the largest light enhancement factor among the examples reported in Table 3. The anisotropic distribution resulted in a lower damping of the LSPR in comparison to the full Pd shell and therefore in higher spatially inhomogeneous electric field at the surface of the Pd dots. According to the authors, the enhanced adsorption of HCOOH on Pd due to the local fields and an increased hot carrier injection rate from the plasmonic to the catalytic metal were crucial to improve the H_2_ production.

Until now, most of the reports utilized Au or Ag as plasmonic metals. However, there is a substantial need to move toward earth abundant metals for sustainable plasmon-driven catalysis. While copper-based systems are the most promising choice due to its inherent resonance within the visible region, the chemical stability of these systems still remains challenging. Luo et al. recently employed Cu–Zn alloys to perform methanol reforming under simulated sunlight illumination.^113^ It was found that the chemical composition of the alloyed nanoparticles influenced the H_2_ production. The largest production (328 × 10^3^ μmol g^–1^ h^–1^) was observed for the Zn_1.3_Cu_98.7_ sample. Both the large production—second best in Table 3—and its promising energy conversion ability reinforce the shift toward earth abundant plasmonic metals.

All these examples shed light on the design of bimetallic hybrid nanocatalysts. The optimization of catalysts is specific to their application, as an excellent catalyst for H_2_ generation from one H_2_ source might not be suitable for H_2_ generation from other H_2_-containing compounds. It has been demonstrated that tailoring the chemical composition and the spatial arrangement of the materials can be beneficial to enhance the interaction between the adsorbate and the catalyst, leading to better catalytic performances. From the reviewed articles included in Table 3, partially coated hybrid bimetallic catalysts seem to be the most promising configurations for sunlight-driven photocatalysis. Compared to fully coated catalysts, these structures only slightly damp the LSPR while still exploiting the interface between the metals for efficient hot carrier transfer.

### Plasmonic Metal–Metal Hybrids for CO_2_ Reduction

In recent years, the ability of PNP to generate energetic carriers when resonantly illuminated has been exploited in multielectron processes such as CO_2_ reduction. However, to our knowledge, to date there appears to be no evidence of plasmonic hybrid bimetallic photocatalysts employed for this reaction. Thus, in this section we will briefly discuss and summarize some relevant monometallic examples in this field (see Table 4).

**Table 4:** Plasmonic Metal–Metal and Plasmonic Metal–Semiconductor Hybrid Systems for CO_2_ Reduction

photocatalyst	light source	major product	temp (°C)	formation rate (μmol g^–1^ h^–1^)	minor products	selectivity (%)	stability (h)	AQE (%)	PE	LE	ref
**PNP–Metal for CO**_**2**_											
Au colloidosomes	lamp (1100 > λ > 400 nm, 1 W cm^–2^)	CH_4_	25 (RT)	1.50 (μmol g^–1^)	–	100	–	–	–	–	(112)
Au-(EMIM-BF_4_)	laser (@532 nm, 10 W cm^–2^)	CH_4_	48	0.76^c^	C_2_H_2_, C_2_H_4_, C_2_H_6_, C_3_H_6_, C_3_H_8_	50	>8	–	–	b	(118)
Au-(EMIM-BF_4_)	laser (@532 nm, 10 W cm^–2^)	CH_4_	48	0.43	C_2_H_2_, C_2_H_4_ C_3_H_6_, C_3_H_8_	50	>40	–	–	b	(119)
Au	Xe lamp (λ > 400 nm, 300 mW cm^–2^)	CH_4_	25	0.068	C_2_H_6_	–	>28	–	–	b	(111)
Ag	theoretical simulations	MeOH	–	DFT calculation	–	–	–	–	–	–	(120)
Ag	laser (@514.5 nm, 0.295 mW μm^–2^)	CO	–	mechanism study by SERS	–	–	–	–	–	–	(121)
Ag	laser (@514.5 nm, 10 mW)	C_4_H_9_OH	–	mechanism study by SERS	–	–	–	–	–	–	(122)

**PNP–Semiconductor for CO**_**2**_											
Au/CdSe-Cu_2_O	300 W Xe lamp (λ > 420 nm)	CO	–	80.3	CH_4_	–	>60	0.4	1.9	b	(123)
Au-TiO_2_(O)	300 W Xe lamp (420 > λ > 320 nm)	CO	25 (RT)	25.6	CH_4_	–	>16	–	5.5	–	(124)
Ag_1_Au_1_/TiO_2_	30 W white LED (λ > 420 nm)	CO	–	0.15	H_2_	–	>15^a^	–	1.6	–	(125)
Au_6_Pd_1_/(101)TiO_2_	300 W Xe lamp	CH_4_	–	12.96	CO, C_2_H_4_ C_2_H_6_	85	–	–	5.2	–	(126)
Ag@TiO_2_	solar simulator (AM1.5G, 300 W)	CH_4_	25	4.93^c^	–	–	>9	0.006	3	–	(127)
BiV O_4_-Au-Cu_2_O	300 W Xe lamp (λ > 420 nm)	CH_4_	–	3.15	CO	–	>20	0.44	5.3	–	(128)
Au/TiO_2_	50 W LED lamp (λ = 530 nm)	CH_4_	3	∼3.00^c^	C_2_H_6_	90	>3^a^	–	2.4	–	(129)

a Experiment duration.

b Negligible product detected without light.

c Calculated or extracted from graph.

Yu et al. reported one of the first examples.^111^ Here, a colloidal suspension of 12 nm AuNPs coated with polyvinylpyrrolidone (AuNP-PVP), was employed to reduce CO_2_. This was possible due to the stability provided by the surfactant when illuminated in long-hour experiments. One of the most striking features of this report was the tunability of the reaction pathway as it was dependent on both the energy of the photons and the photon flux. As can be seen in Figure 2e, when illuminated with visible light (λ > 400 nm, 300 mW cm^–2^), the AuNPs promoted the formation of both CH_4_ and ethane (C_2_H_6_). To understand the mechanism, the researchers further studied the role of the plasmon and interband transitions in the reaction pathway by varying both the wavelength and power. When using green photons matching the LSPR (λ = 532 nm), the AuNPs only produced CH_4_ irrespective of the power. However, when the incident photons were more energetic (λ = 488 nm), AuNPs not only were able to produce CH_4_, but also promoted C–C coupling at high powers (>300 mW cm^–1^), resulting in C_2_H_6_ production. The possibility to conduct a 14 e^–^ (C_2_H_6_) instead a 8 e^–^ (CH_4_) reduction was attributed to longer lifetimes of energetic charge carriers when exciting the interband transitions compared to the hot carriers created by means of LSPR. Hybrid plasmonic systems can potentially decrease charge recombination and therefore could allow the production of C2+ products by plasmon excitation of bimetallic systems in the future. Dhiman et al. investigated the influence of hotspots on the methanation of CO_2_ using dendritic plasmonic colloidosomes (DPC) (see Figure 2f).^112^ Clustering the NPs allowed the utilization of not only the visible, but also the NIR region of the solar spectrum. Interestingly, they found that the performance of the system was dependent on the Au load and the strength of the hotspots created within the interparticle gaps. Specifically, only the sample with ∼8 nm AuNPs (DPC-C4, four growing steps) enabled the reduction of CO_2_ to generate CH_4_, as shown in Figure 2f. The CH_4_ production of 1.50 μmol g^–1^ h^–1^ is one of the highest in Table 4, although it is worth mentioning that the experiments were conducted at 85 °C. Conversely, colloidosomes with either smaller or larger nanoparticles were not able to reduce the CO_2_ neither to CO nor CH_4_. The performance of the 8 nm AuNPs colloidosomes was attributed to the synergistic effects of the optimized electromagnetic and thermal hotspot coupled to the hot carriers transfer for Au surfaces to adsorbed CO_2_.

It has been demonstrated that multielectron processes can be driven by PNPs, although there is still plenty of room to explore toward the fabrication of plasmonic bimetallic photocatalysts for CO_2_ reduction. Proper engineering of the hybrid should focus on ways to increase the pumping of hot carriers toward the active material. Also, the strong adsorption of CO on metallic surfaces must be taken into account for proper materials selection. This phenomenon should not be underestimated as it leads to the inactivation of the active sites. An emerging way to engineer the materials for C–C coupling is to create nanometer gaps between the plasmonic and catalytic metal surfaces. In this type of catalyst, the reduction of CO_2_ at the surface of the PNP can create intermediate-enriched environments nearby the catalytic metal surface. This is usually referred to as in-tandem reactors.

## Plasmonic Metal–Semiconductor

**Figure 3:**
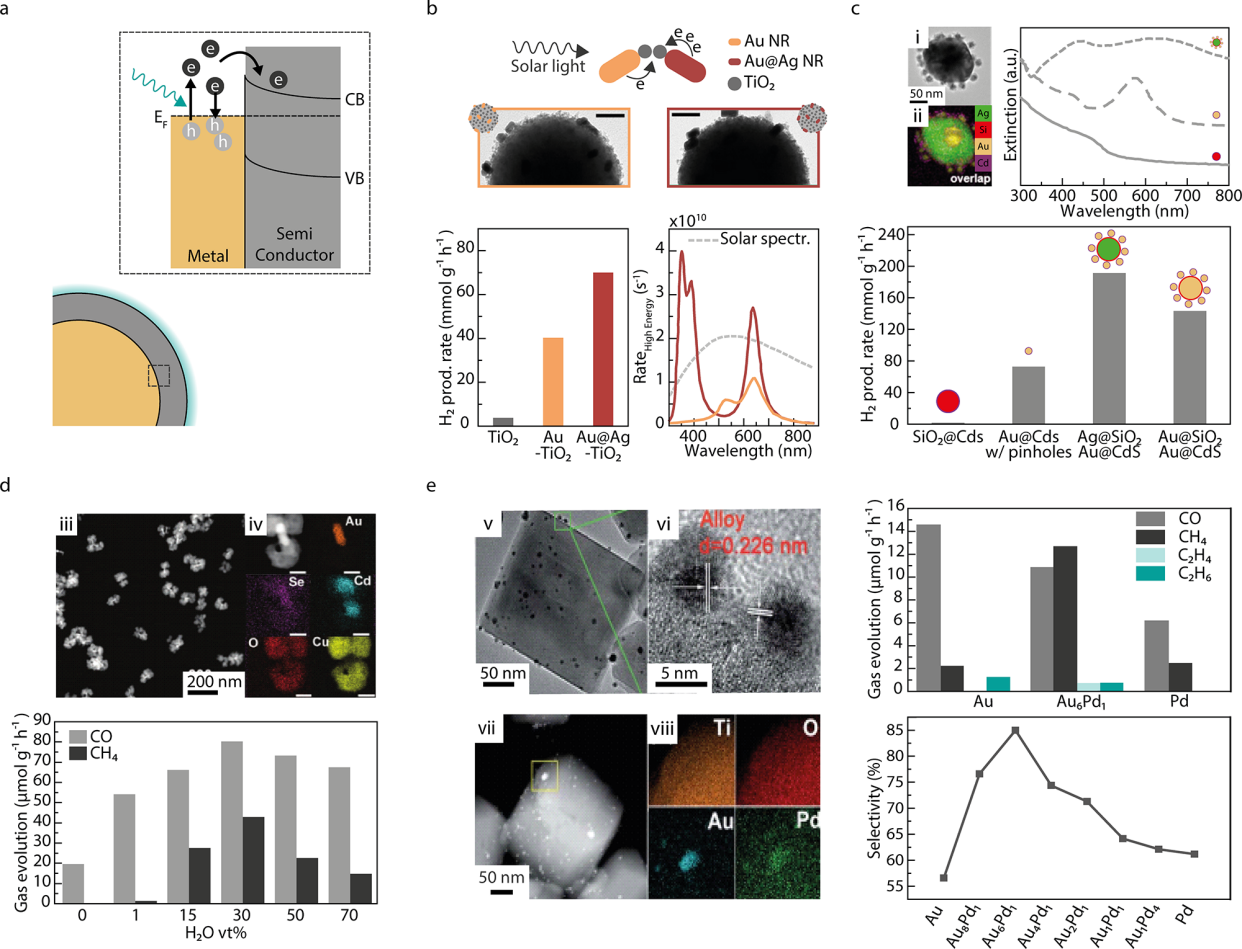
Plasmonic metal–semiconductor hybrids. (a) Schematic representation of charge transfer at the Schottky barrier of a metal–semiconductor interface. (b) Top: TEM images of both AuNR-TiO_2_ and Au@Ag NR-TiO_2_ supported on SiO_2_ (scale bars 100 nm). Bottom: photocatalytic performance of the hybrid systems toward FA dehydrogenation and calculated hot electrons generation rates for the Au and Au@Ag NRs under sunlight illumination. (c) Top: TEM (i), corresponding EDS (ii), and optical characterization of Ag@SiO_2_/Au@CdS hybrid. Bottom: performances of a set M-SC hybrids for optimized HER. (d) Top: HAADF (iii) and EDS images (iv, scale bars 20 nm) of Au/CdSe-Cu_2_O used in CO2RR. Bottom: performance of the hybrid in CO2RR as a function of H_2_O percentage in the reaction mixture. (e) Left: TEM (v), HR-TEM (vi), HAADF (vii), and corresponding EDS (viii) images of (101) TiO_2_-supported Au_1_Pd_1_ alloy NPs. Top-right: CO2RR for Au, Pd, and alloyed AuPd (stoichiometry ratio Au:Pd 6:1). Bottom-right: selectivity toward the hydrocarbons varying the chemical composition of Au–Pd alloy NPs. (a,b) Adapted with permission from ref. (131). Copyright 2020 American Chemical Society. (c) Adapted with permission from ref. (132). Copyright 2021 American Chemical Society. (d) Adapted with permission from ref. (123). Copyright 2020 Wiley-VCH. (e) Adapted with permission from ref. (126).Copyright 2019 Royal Society of Chemistry.

The charge transfer from a plasmonically excited metal nanoparticle to a semiconductor, schemed in Figure 3a, has been beneficial for increasing the photocatalytic production of solar fuels. The pursuit of an optimized hybrid photocatalyst has led to the combination of PNPs with several semiconductors such as TiO_2_, CdS, and Cu_2_O, among others. Recent examples of these types of hybrid materials are summarized in Table 4 and Table 5, as well as the performance metrics defined in the introduction. In the following, selected examples for both H_2_ generation and CO_2_ reduction will be discussed.

**Table 5:** Plasmonic Metal–Semiconductor Hybrid Systems for H_2_ Generation

photocatalyst	light source	reaction	temp (°C)	formation rate (μmol g^–1^ h^–1^)	stability (h)	AQE (%)	PE	LE	ref
**PNP–Semiconductor for H**_**2**_									
Pt/TiO_2_-HA	300 W Xe lamp (λ > 400 nm)	H_2_O reduction (EtOH)	–	356 × 10^3^	>56	0.23 (@550 nm)	–	–	(130)
Ag@SiO_2_/ Au@CdS with pinholes	0.35 W LED strips (λ ≥ 400 nm)	H_2_O reduction (Na_2_SO_3_/Na_2_S)	15	191 × 10^3^	–	–	7346	–	(132)
Au@SiO_2_/ Au@CdS with pinholes	LED strips (λ ≥ 400 nm)	H_2_O reduction (Na_2_SO_3_/Na_2_S)	15	143 × 10^3^	–	–	5500	–	(132)
Ag@SiO_2_@CdS@Au	LED strips (λ ≥ 400 nm, 0.198 mW cm^–2^)	H_2_O reduction (Na_2_SO_3_/Na_2_S)	12	130 × 10^3^	–	0.35 (@475 nm)	3.3	–	(133)
Au@CdS with pinholes	LED strips (λ ≥ 400 nm)	H_2_O reduction (Na_2_SO_3_/Na_2_S)	15	75000	–	–	2885	–	(132)
Au@Ag nanorods/TiO_2_	300 W Xe lamp (λ ≥ 350 nm)	FA dehydrogenation	35	60000	>1^a^	–	b	–	(131)
AuNR/TiO_2_	300 W Xe lamp (λ ≥ 350 nm)	FA dehydrogenation	35	40000	>1^a^	–	b	–	(131)
Ag@SiO_2_@CdS	white lamp (198 W cm^–2^)	H_2_O reduction (Na_2_SO_3_/Na_2_S)	12	38000	–	–	75		(133)
Au@CdS, pinhole-free	LED (λ ≥ 350 nm)	H_2_O reduction (Na_2_SO_3_/Na_2_S)	15	30000	–	–	21.4	–	(132)
Au@CdS	300 W Xe lamp (λ ≥ 420 nm)	H_2_O reduction (Na_2_SO_3_/Na_2_S)	20	24000	>20	48^d^	240	–	(135)
AuNP chain-Zn_0.67_CdS_0.33_	Xe lamp (λ ≥ 420 nm, 300 W cm^–2^)	H_2_O reduction (Na_2_SO_3_/Na_2_S)	15	16420	>40	54.6 (@420 nm)	3.3	–	(136)
TiO_2_-Au nanofibers	300 W Xe lamp (λ ≥ 420 nm)	H_2_O reduction (MeOH)	–	12440	>3^a^	5.11 (@400 nm)	10	–	(137)
AuNPs/TiO_2_ nanorod	300 W Xe lamp	H_2_O reduction (EtOH)	25	7100	>30	–	57	–	(134)
AuNPs@TiO_2_-Pt	300 W Xe lamp (λ ≥ 420 nm)	H_2_O reduction (MeOH)	–	3100	>4^a^		–	–	(138)
AgNPs/TiO_2_ nanorods	150 W Xe lamp	H_2_O reduction (MeOH)	–	740	>45	–	6.2	–	(139)
Pt-decorated Au-TiO_2_ nanodumbbell	300 m W Xe lamp (λ ≥ 420 nm)	H_2_O reduction (MeOH)	–	350	>10	–		–	(140)
Au@CdS/ZnO	LED lamp (λ = 640 nm, 3.4 mW cm^–2^)	H_2_O reduction (MeOH)	25	79	>200	0.24 (@640 nm)	–	c	(142)
Au nanostars@c-TiO_2_	Xe lamp (λ ≥ 420 nm, 100 mW cm^–2^)	H_2_O reduction (MeOH)	–	77	>17	2.3 (@650 nm)	–	–	(143)
SiO_2_/AuNPs@TiO_2_	300 W Xe lamp (λ ≥ 420 nm)	H_2_O reduction (MeOH)	–	52	>5^a^	–	–	–	(144)
3D array Au-TiO_2_-Pt	Xe lamp (λ ≥ 420 nm, 650 mW cm^–2^)	H_2_O reduction (MeOH)	15	43	>9	0.43 (@540 nm)	b	–	(145)
Au nanorods/TiO_2_ dumbbell	300 W Xe lamp	H_2_O reduction (MeOH)	30	12	–	–	b	–	(146)
Au nanostars@TiO_2_	150 W Xe lamp (630 > λ > 420 nm)	H_2_O reduction (MeOH)	40	7.9	>0.33^a^	–		–	(141)
Fe_3_O_4_-Au-CdS	Xe lamp (λ ≥ 420 nm)	H_2_O reduction Na_2_SO_3_/Na_2_S)	65	106 μmol h^–1^	>12	–	–	–	(147)
SCN^–^-functionalized AgNPs/TiO_2_	300 W Xe lamp (λ ≥ 320 nm)	H_2_O reduction (MeOH)	–	45 μmol h^–1^	>15	–	27.5	–	(148)
Fe_3_O_4_-CdS-Au	Xe lamp (λ ≥ 420 nm)	H_2_O reduction (Na_2_SO_3_/Na_2_S)	–	13 μmol h^–1^	>30	–	1.33	–	(149)

a Experiment duration.

b Negligible product detected without plasmonic material.

c Neglibible product detected without light.

d QY of hot electron injection from transient absorption spectra.

### Plasmonic Metal–Semiconductor Hybrids for H_2_ Generation

As shown in Table 5, more than 60% of the hybrids for plasmon assisted photocatalytic H_2_ production utilized TiO_2_ as the semiconductor component, owing to its unique photochemical and photoelectrochemical properties. Herein, the best photocatalyst for hydrogen production was realized by Qin et al.^130^ They designed and employed an efficient photocatalyst for plasmon-enhanced visible light based on Pt nanoparticles (PtNPs) supported on TiO_2_ photocatalysts (Pt/TiO_2_). While anatase-structured TiO_2_ is only active in the UV, the inclusion of 100 nm plasmonic PtNPs allowed to extend the photoactivity of the hybrid to the visible. The improved H_2_ formation rate of 356 × 10^3^ μmol g^–1^ h^–1^, the highest among all the reviewed metal–semiconductor examples, was a result of transferring hot electrons from Pt to the conduction band of TiO_2_. To understand the underlying mechanism, the PtNPs were replaced by AuNPs (50 nm). Unexpectedly, this system did not produce as much H_2_ as its Pt analogue. While the excitation of the plasmon in both metals enabled the injection of energetic electrons into the conduction band of TiO_2_, their different Schottky barrier strongly affected the photocatalyic performance. In short, the higher Schottky barrier of the Pt-TiO_2_ heterojunction of ϕ^Pt^ = 1.7 eV (compared to ϕ^Au^ = 0.9–1.0 eV with Au) inhibited the recombination of the injected electrons in TiO_2_ with the holes in the metal. This led to an extended lifetime and larger extraction toward H_2_ production. Remarkably, in addition to its high production, it is also one of the most stable hybrids of this section (56 h).

Negrin-Montecelo et al. published an important study on the critical influence of morphology and composition of the plasmonic component in PNP–TiO_2_ nanostructures.^131^ Herein, the authors interfaced both Au nanorods (NR) and core–shell Au@Ag NR with TiO_2_ and tested them toward the dehydrogenation of HCOOH (Figure 3b). In their experiments, it was found that by coating a AuNR with a Ag shell, the H_2_ production was increased from roughly 36.5 × 10^3^ μmol g^–1^ h^–1^ to 64.9 × 10^3^ μmol g^–1^ h^–1^. As expected, TiO_2_ on silica beads was not able to trigger the reactions when illuminated with photons below the energy of the bandgap. This reinforces the role of the plasmonic component as the photosensitizer material. Theoretical calculations revealed that the Au@AgNRs were able to create both more and higher energy electrons than the bare AuNRs, facilitating the injection of hot electrons into the conduction band of the semiconductor.

Recently, CdS has emerged as a promising semiconductor to be exploited in the generation of H_2_, as seen in the first five examples listed in Table 5. Yang et al. synthesized hybrid dual plasmonic antenna consisting of partially CdS-coated Au satellites assembled on SiO_2_-coated Ag nanoparticles (Ag@SiO_2_/Au@CdS with pinholes).^132^ Panels i and ii of Figure 3c show the spatial arrangement of the metals. When tested for the HER, this structure yielded one of the highest performances for this type of PNP–SC hybrids (191 × 10^3^ μmol g^–1^ h^–1^, Figure 3c). It was possible to improve the formation rate by 7346 times, as evidenced by the plasmonic enhancement factor. The combination of these three materials resulted in an optical response extending from the blue to the red of the visible region. The inherent absorption of the CdS was enhanced due to the overlap with the Ag resonance, and hot carriers were pumped into the conduction band of the semiconductor by the Au satellite, leading to a notable improvement in the performance. Finally, when the Ag antenna was replaced by a Au optical antenna in the hybrid, the performance was slightly inferior (143 × 10^3^ μmol g^–1^ h^–1^). The authors attributed this result to the poor overlap between the Au LSPR and the CdS absorption band in the blue region, as shown in the extinction spectra in Figure 3c.

In conclusion, tremendous advances have been made in the combination of PNP with semiconductors toward the generation of H_2_. Due to its high photoactivity, low cost as well as excellent chemical stability, TiO_2_ is the most used semiconductor material in hybrid plasmonic semiconductor systems so far. Nevertheless, novel hybrids with promising performances are arising. Challenges to be addressed in the near future are the susceptibility of CdS to corrosion under light illumination^150^ and the high recombination rate of carriers of ZnO,^151^ limiting the chemical stability and catalytic activity, respectively.

### Plasmonic Metal–Semiconductor Hybrids for CO_2_ Reduction

The creation of an heterojunction between metals and semiconductor is a promising strategy to suppress the HER thereby enhancing the photocatalytic CO_2_RR. Both the activity and selectivity can be tuned to favor a desired product of the reaction. Recent reports on the application of these hybrids are summarized in Table 4.

One of the most remarkable examples in terms of selectivity and production of carbon-based products was recently reported by Wang et al.^123^ The authors synthesized Au/CdSe-Cu_2_O dumbbell nanorods (see Figure 3d, panels iii, iv, v) which were utilized for CO_2_RR under visible illumination (λ > 420 nm) at different H_2_O %wt in the reactant solution. Throughout the tests, H_2_ was never detected, and CO as well as more electron-demanding products such as CH_4_ were produced instead. As shown in Figure 3d, the production reached maximum values of 80.1 and 42.9 μmol g^–1^ h^–1^ for CO and CH_4_, respectively. The water provided the H atoms for the 8e^–^/8H^+^ reduction of CO_2_ to CH_4_. By combining Au with these semiconductors, a plasmonic enhancement of 1.92 was achieved. The results obtained for this hybrid (Au/CdSe-Cu_2_O dumbbell nanorods) were explained by the charge transport in the materials. In short, all three components can be excited simultaneously under visible illumination, and as a result, the photogenerated electrons accumulate in Cu_2_O due to the recombination of its respective holes with electrons injected from Au and CdSe.

Chen et al.^126^ reported a systematic study where the reactivity and selectivity of TiO_2_ with (101) facets were found to increase with the photoreduction of Au–Pd nanoalloys of different ratio compositions. By anchoring the Au–Pd co-catalyst on TiO_2_ (101), the Au–Pd alloys with the stoichiometric ratio of 6:1 provided a maximum selectivity toward hydrocarbons of 85% in the CO_2_RR under visible light illumination (see Figure 3e). In addition to the detection of CH_4_, which was the main product (12.9 μmol g^–1^ h^–1^), ethane (C_2_H_6_) and ethylene (C_2_H_4_) were detected, evidencing the capability to perform C–C coupling. While the production of CH_4_ was still observed when only Pd was reduced on top of TiO_2_ (101), the inclusion of Au led to a notable 5.2-fold increase.

The application of hybrids toward the reduction of CO_2_ is still under development, and so far only a few combinations of metals and semiconductors have been explored. The majority of the examples listed in Table 4 employed both Au and TiO_2_ NPs, and only one out of seven used Ag as the plasmonic component. It is also worth mentioning that in only one report the photocatalyst was tested under simulated solar illumination conditions. It is necessary to aim for sustainable ways to carry out these processes. In this regard, cheap semiconductors such as TiO_2_ and plasmonic metals (e.g., Al or Cu) could emerge as candidates. Cu-based systems are promising especially for C2+ products and because of the abundancy of Cu. However, to our knowledge, they have not been explored yet. At last, the design of novel heterostructures should be carefully addressed to maximize the hot carriers injection in the active centers, as well as the lifetime of hot carriers created by means of plasmon excitations. This will lead to higher efficiencies and will bring the hybrids one step closer to industrial applications.

## Plasmonic Metal–Perovskite

**Figure 4:**
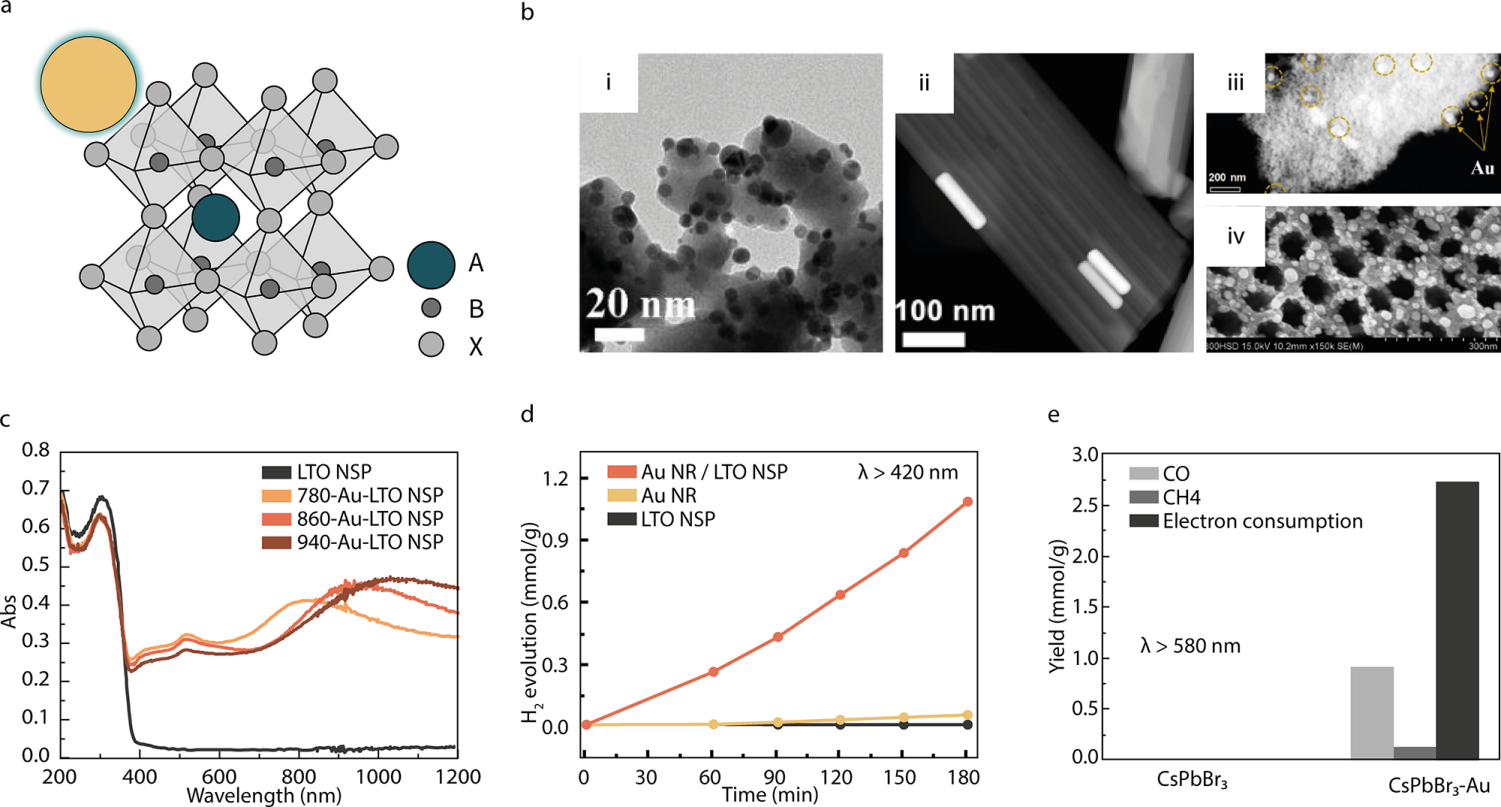
Plasmonic metal–perovskite hybrids. (a) Schematic representation of a plasmonic–perovskite hybrid system. (b) Exemplary architectures of hybrid structures: (i) TEM image of CsPbBr_3_ nanocubes with Au nanoparticles, (ii) TEM image of La_2_Ti_2_O_7_ (LTO) nanosteps with Au nanorods, (iii) TEM image of LaFeO_3_ nanostructure with Au nanoparticles, and (iv) SEM image of SrTiO_3_ nanotube arrays with Ag nanoparticles. (c) By employing Au Nanorods with different resonances, the absorption of the hybrid Au–LTO structure (shown in (ii)) can be tuned and extended across the entire solar spectrum. (d) Photocatalytic H_2_ production of the components Au and LTO and the Au–LTO hybrid structure. (e) Photocatalytic CO_2_ reduction of CsPbBr_3_ nanocubes and the hybrid structure with Au (shown in (i)), excited with energies below the band gap of the perovskite. (b-i,e) adapted with permission from ref. (155). Copyright 2020 Elsevier. (b-ii,c,d) adapted with permission from ref. (156). Copyright 2018 American Chemical Society. (b-iii) adapted with permission from ref. (70). Copyright 2019 Elsevier. (b-iv) Adapted with permission from ref. (96). Copyright 2011 Elsevier.

The synergistic effects of the combination of the light-harvesting properties of PNPs with the optoelectric and catalytic properties of perovskite materials have resulted in the investigation of various geometrical and material arrangements (see Figure 4b). In Table 6, the hybrids that have been tested toward H_2_ generation and CO_2_ reduction are summarized using the metrics specified in the introduction. In the following, examples with noteworthy properties in terms of formation rate, material composition, or operational wavelength range will be discussed. To our knowledge, most systems studied to date for solar fuels production are based on perovskite oxides. Halide perovskites are another promising set of materials due to the fact that their tunable absorption across the visible can be enhanced by the local field enhancement of PNPs. This has been shown to be useful in photovoltaic applications.^152−154^ However, the current bottleneck for photocatalytic applications is their instability. Under external stimuli such as moisture, halide perovskites are easily decomposed, which is detrimental for many catalytic applications like water-splitting. This may be one of the reasons why there is no demonstration of hydrogen production on halide perovskites yet, and that the first demonstration of CO_2_ reduction in such a system was published in 2020.^155^

**Table 6:** Plasmonic Metal–Perovskite Hybrid Systems for H_2_ Generation and CO_2_ Reduction

photocatalyst	light source	reaction	temp (°C)	formation rate (μmol g^–1^ h^–1^)	stability (h)	AQE (%)	PE	LE	ref
**PNP–Perovskite for H**_**2**_									
Ag/AgTaO_3_-SrTiO_3_	500 W Hg lamp (λ > 250 nm)	H_2_O reduction (Na_2_SO_3_)	–	2150^c^	>9	2.02%	–	–	(161)
Al/BaTiO_3_	solar simulator (AM1.5G, 100 mW cm^–2^)	H_2_O reduction (MeOH)	–	∼1635^c^ (∼3285^e^)	>30	–	6.8^c^	d	(160)
Au/Pr_0.5_(Ba_0.5_Sr_0.5_)_0.5_Co_0.8_Fe_0.2_O_3_	300 W Xe lamp (300 mW cm^–2^)	H_2_O reduction (CH_2_O)	–	1618	>8	–	∼540	–	(165)
Au/La_2_TiO_7_/BP	Xe lamp (λ > 420 nm)	H_2_O reduction (MeOH)	–	740	>12	–	b	–	(162)
Au/SrTiO_3_/TiO_2_	300 W Xe lamp (λ > 320 nm)	H_2_O reduction (MeOH)	–	467.3	>8^a^	–	75	–	(163)
Au/La_2_TiO_7_	Xe lamp (350 mW cm^–2^, λ > 420 nm)	H_2_O reduction (MeOH)	25 (RT)	340	>24	1.4% (@900 nm)	–	–	(156)
Ag/SrTiO_3_	300 W Xe lamp (λ > 420 nm)	H_2_O reduction (MeOH)	6	264.5	>16	–	b	d	(157)
Au/LaFeO_3_	solar simulator (AM1.5G, 300 mW cm^–2^)	H_2_O reduction (Na_2_S/Na_2_SO_3_)	5	202	>6^a^	–	∼2	–	(70)
Ag/KTaO_3_	solar simulator (300 W)	H_2_O reduction (MeOH)	<5	185.6^c^	>7^a^	–	1.9^c^	–	(158)
Ag/AgTaO_3_	300 W Xe lamp (λ > 420 nm)	H_2_O reduction (C_2_H_2_O_4_)	20	100	>40	–	5	–	(166)
Ag/AgTaO_3_-SrTiO_3_	500 W Hg lamp (λ > 400 nm)	H_2_O reduction (Na_2_SO_3_)	–	58	>9	0.11%–	–	–	(161)
Au/LaCoO_3_	500 W Xe lamp (300 mW cm^–2^)	H_2_O reduction (CH_2_O)	5	42	>1.5^a^^,^^c^	–	2.6^c^	–	(159)
Ag/KTaO_3_	300 W Xe lamp (visible)	H_2_O reduction (MeOH)	<5	25.94	>7^a^	–	b	–	(158)
Ag/NaTaO_3_	300 W Xe lamp	H_2_O reduction (MeOH)	<5	3.54	>7^a^	–	2.1^c^	–	(158)
Au/SrTiO_3_:Nb	Xe lamp	H_2_O reduction (chemical bias)	–	7 nmol h^–1^	>48	4.4 × 10^–4^% (@600 nm)	pH bias reduced	–	(164)


photocatalyst	light source	major product	temp (°C)	formation rate (μmol g^–1^ h^–1^)	minor products	selectivity (%)	stability (h)	AQE (%)	PE	LE	ref
**PNP–Perovskite for CO**_**2**_											
Au/CsPbBr_3_	300 W Xe lamp (λ > 420 nm)	CO	–	∼10.44^c^	CH_4_		–	–	∼2.1^c^	–	(167)
Ag/SrTiO_3_	300 W Xe lamp (λ > 420 nm)	CO	5	80.24 μmol g^–1^	H_2_, CH_4_	–	>16	–	–	–	(157)
Au/CsPbBr_3_	100 W Xe lamp (λ > 420 nm)	CO	–	∼10 μmol g^–1^^c^	CH_4_	∼76%^c^	–	–	∼2.1^c^	–	(155)
Au/CsPbBr_3_	100 W Xe lamp (λ > 580 nm)	CO	–	∼1 μmol g^–1^^c^	CH_4_	∼85%^c^	–	–	b	–	(155)

a Experiment duration.

b Negligible product detected without plasmonic material.

c Values extracted from the graph or calculated.

d Negligible product detected without light.

e Piezo-photocatalysis (additional mechanical stimuli).

### Plasmonic Metal–Perovskite Hybrids for H_2_ Production

Plasmon–perovskite hybrids investigated toward H_2_ production recently are shown in Table 6. Out of the eight different pervoskite oxide materials shown in Table 6, seven^70,156−163^ are wide band gap semiconductors which require UV light for optical excitation. Extending the light absorption into the visible region with plasmonic hybrids is a promising strategy to increase the solar-to-fuel conversion efficiency (illustrated in Figure 4c). To probe whether hybridizing such a wide bang gap perovskite with a plasmonic material allows for photocatalytic H_2_O reduction using only visible light, Wang et al. synthesized SrTiO_3_ nanocubes both with and without AgNPs.^157^ When illuminated with a 300 W Xe lamp filtered to wavelengths longer than 420 nm, the SrTiO_3_ nanocubes alone predictably did not produce any hydrogen. The Ag/SrTiO_3_ nanocomposite, however, was able to catalyze H_2_ production and reached a formation rate of 264.5 μmol g^–1^ h^–1^. Only one other perovskite hybrid system was reported to reach a comparable high formation rate at wavelengths longer than 420 nm (see Table 6). This hybrid photocatalyst investigated by Cai et al. not only allowed the utilization of visible light but extended the usable range into the infrared (IR).^156^ This was achieved by a hybrid system consisting of Au nanorods (AuNRs) on lanthanum titanium oxide (La_2_Ti_2_O_7_, LTO) nanosteps, depicted in panel ii of Figure 4b. Figure 4c shows how the absorption peak shifts when AuNRs of different plasmon resonances are incorporated in the system. It illustrates how the geometry of the PNP can be used to tune the absorption of a plasmon-perovskite hybrid system across the solar spectrum. The system reached its maximum apparent quantum efficiency (AQE) of 1.4% at 920 nm, the wavelength coinciding with the longitudinal plasmon resonance of the employed AuNRs. While neither the AuNRs nor LTO individually produced a significant amount of H_2_ when illuminated with visible light, the combined system did, as can be seen in Figure 4d. The synergistic effect arose because the low energy photons could not photoexcite the system without the AuNRs due to the large bandgap of LTO and, on the other hand, the hot electrons generated in the AuNRs under resonant illumination could only be extracted effectively through the interface with LTO.

Most plasmonic systems are restricted to noble metals, making large-scale industrial applications expensive. This has resulted in a search for cheaper plasmonic alternatives. As far as we know, the only example of a plasmon–perovskite hybrid for H_2_ production utilizing non-precious materials was published by Guo et al.^160^ By combining barium titanate (BaTiO_3_) with aluminum, a plasmonic H_2_-production enhancement factor of 6.8 was achieved, showing the potential of non-noble metals. Additionally, the use of BaTiO_3_ allows for the combination of plasmonics with piezoelectronics, where mechanical stimuli significantly enhance the photogenerated carrier separation and transfer, further increasing the H_2_-production rate by a factor of ∼2.

The Al/BaTiO_3_ hybrid as well as eight^70,159,164,165^ out of the 15 systems shown in Table 6 were illuminated using UV–visible–IR light, optically exciting both components. One effect that should not be neglected when discussing plasmonic hybrid systems is the effect the metal has on the system, even when the plasmon is not excited. When a semiconductor is excited above the band gap, the metal can act as a charge trap, increasing carrier lifetimes. Xu et al.^158^ probed this by testing the H_2_ production on KTaO_3_ and NaTaO_3_ decorated with AgNPs for both visible–NIR and UV–visible–NIR excitation. In both cases, the addition of AgNPs increased the formation rates. However, the H_2_ formation rates of the composites under UV–visible–NIR light were higher than under purely visible–NIR light. The enhancement in H_2_ production by the addition of AgNPs is therefore described as a combination of AgNPs acting as co-catalysts by increasing the charge separation of electron–hole pairs generated in the perovskite by excitation with UV light, as well as absorption in the visible and subsequent hot electron transfer from the AgNPs. The goal is to eventually use the full solar spectrum spanning from the UV to the IR for the photocatalytic production of H_2_, and hence all synergistic effects can and should be exploited. However, at the current stage, still far from industrial applications, systematic wavelength-dependent studies reporting not only on production rates but also the quantum efficiencies, like the work of Cai et al.^156^ and Zhong et al.,^164^ are needed to understand the underlying mechanisms and design catalysts accordingly.

The studies conducted to date and presented above as well as in Table 6 show that plasmon–perovskite hybrids can improve H_2_ production rates compared to the pristine perovskites by enhancing the absorption across the solar spectrum and improving charge carrier dynamics. While they are limited to perovskite oxides and H_2_ generation from water, these findings show the potential of exploring also other H_2_ generating reactions and eventually perovskite types like perovskite halides.

### Plasmonic Metal–Perovskite Hybrids for CO_2_ Reduction

The combination of a plasmonic material with a perovskite for photocatalysis has shown to be beneficial not only for H_2_ production but can also enhance other photocatalytic processes like dye or isopropanol degradation,^168−171^ or the removal of algae.^172^ However, as far as we know, there are only three publications on photocatalytic CO_2_ reduction using plasmon-perovskite hybrids. They are shown in Table 6. The first, by Wan et al. was briefly discussed above, as the same catalyst was also investigated toward H_2_ production.^157^ As expected, no CO_2_ reduction occurred under visible–NIR light for the SrTiO_3_ nanocubes. For the Ag/SrTiO_3_ hybrid, three products were detected: CO, H_2_, and CH_4_. As commonly reported for plasmonic photocatalysis with perovskite oxides, the enhanced photocatalytic reduction activity is attributed to improved visible light absorption and efficient charge separation. The competing HER increases with increasing Ag loading, which is explained by an increase in the recombination of charge carriers. The selectivity toward CO formation could be optimized by choosing an intermediate amount of Ag.

Liao et al. published the first halide perovskite-based plasmonic photocatalyst in 2020. In their study, the experiments were not carried out in an aqueous solution but in a mixture of acetonitrile and isopropanol. CsPbBr_3_ nanocubes were decorated with AuNPs (see panel i in Figure 4b) and tested toward their CO_2_ reduction performance with both visible–NIR light (λ > 420 nm) and light below the bandgap of CsPbBr_3_ (λ > 580 nm).^155^ The results are in agreement with the findings of Xu et al.^158^ (discussed above for H_2_ production), where the increase in activity due to the presence of the AuNPs could be attributed to both charge transfer from the perovskite to Au and charge transfer from Au to the perovskite, dependent on which entity was optically excited. For full visible–NIR light illumination, the introduction of AuNPs yields a 3.2-fold enhancement. The results for below bandgap excitation with wavelengths longer than 580 nm can be seen in Figure 4e. As expected, no CO_2_ reduction can be detected for the perovskite alone. The Au/CsPbBr_3_ extends the usable wavelength range further into the visible, as indicated by the CO_2_ conversion rate. Since then, these findings have been supported by Tang et al., who used a macroporous CsPbBr_3_ framework combined with AuNPs^167^ instead of Au/CsPbBr_3_ nanocubes. With the first application of plasmonic perovskite hybrids toward the reduction of CO_2_ published in 2019, the current systems lack in efficiency but are proof that there is a lot of room to explore. Thus far, the main product is CO. Whether a higher selectivity toward C2+ products can be achieved remains to be answered.

The recent publications on hybrid plasmon perovskite structures reviewed here show that the combination of plasmonics and perovskites is a promising strategy for the photocatalytic production of solar fuels. The systems explored so far are mostly limited to perovskite oxides. Their abundance makes them attractive materials for large-scale photocatalysis. To enhance their solar catalytic performance while keeping costs low, the combination with abundant plasmonic materials like aluminum will be an interesting and potentially fruitful route to explore. Apart from the opportunities for perovskite oxides, halide perovskites for plasmon-assisted photocatalytic applications are another promising field. Their tunable absorption across the visible can be enhanced by the local field enhancement of PNPs, which has been shown in photovoltaic applications.^173^ The current bottleneck for photocatalytic applications is their instability. Many efforts are currently made to improve their stability^174^ and decrease their environmental toxicity,^175^ which will allow further exploration of their combination with plasmonic materials for the production of solar fuels.

## Plasmonic Metal–2D

**Figure 5:**
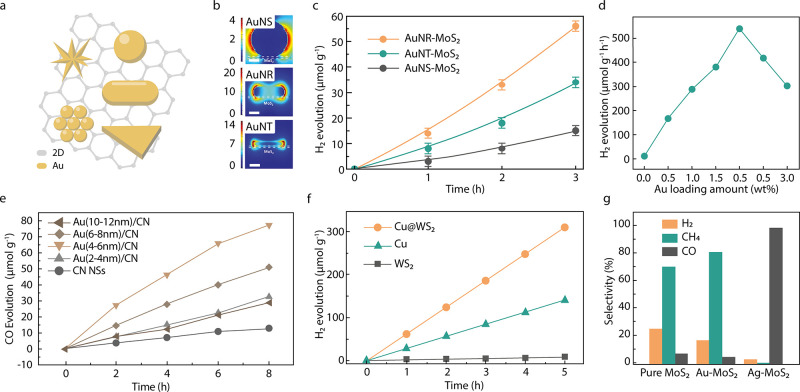
Plasmonic metal–2D hybrids. (a) Schematic representation of a 2D material loaded with plasmonic metal nanostructures of various shapes, sizes, and compositions. (b–g) illustrate the effect on catalytic activity of shape, loading amount, size, hybrid vs single material, and plasmonic material, respectively. (b) FDTD simulations on electromagnetic fields of AuNS–MoS_2_, AuNR–MoS_2_, and AuN–TMoS_2_ hybrid structures. Scale bars are 20 nm. Effects of (c) Au shape (d) and Au loading ratio (in wt%) on photocatalytic H_2_ evolution. (e) Effect of AuNP size on the CO production rate. (f) H_2_ evolution rate for both single plasmonic (Cu) and 2D (WS_2_) photocatalyst and the hybrid structure. (g) Product selectivity of CO_2_ reduction using a Ag–2D and Au–2D hybrid catalyst. (b, c) Adapted from ref. (181). Copyright 2017 Royal Society of Chemistry. (d) Adapted from ref. (182). Copyright 2018 Royal Society of Chemistry. (e) Adapted from ref. (185). Copyright 2019 Elsevier. (f) Adapted from ref. (186). Copyright 2018 Wiley. (g) Adapted from ref. (74). Copyright 2020 Elsevier.

This section covers metal–2D hybrid-based solar H_2_ production and CO_2_ reduction (see Figure 5a). Conventionally, 2D materials refer to single-crystalline materials that consist of single or several layers of atoms. Strictly speaking, the electron movement within the material should be only confined along the perpendicular direction of the 2D plane.^176^ These atomically thin crystalline 2D materials are commonly obtained by mechanical or chemical exfoliation, and due to this nature, their reproducibility is highly limited. Some argue that nanosheets and -plates can also be referred to as 2D materials as long as a certain degree of quantum confinement along the thickness direction is maintained.^177^ These types of materials are studied intensively in recent years due to their mass producibility.^178,179^ Therefore, in the following, nanosheets and nanoflakes will also be categorized as 2D materials. Tables 7, 8, and 9 summarize some of the recent advances on metal–2D hybrid systems. This section aims to cast insight into tuning and controlling the properties of plasmonic metal–2D hybrids for photocatalytic applications. As can be seen from the tables, overall, by combining plasmonic metal nanostructures and 2D materials, the photocatalytic performances of the hybrid systems have improved compared to their 2D single-component counterparts.

**Table 7:** Plasmonic Metal–2D for H_2_ Generation (1/2)

photocatalyst	light source	reaction	temp (°C)	formation rate (μmol g^–1^ h^–1^)	stability (h)	AQE (%)	PE	LE	ref
**PNP–2D for H**_**2**_									
AgPd/g-C_3_N_4_	Xe lamp (λ ≥ 420 nm, 35 mW cm^–2^)	FA dehydrogenation (HCOONa)	30–40	2.32 × 10^6^ c	>20 min^a^	27.8 (@400 nm)	–	–	(187)
Pd/Mo_*x*_W_1–*x*_O_3–*y*_	Xe lamp (λ > 420 nm, 63.5 mW cm^–2^)	NH_3_BH_3_ dehydrogenation	20	∼8 × 10^5^ c	>50 min	–	∼5	2.3	(188)
Cu/WS_2_	solar simulator (AM1.5G, 300 W, 100 mW cm^–2^)	H_2_O reduction	26	64000	>30	15.6^c^ (@500 nm)	40	–	(186)
AuPd/rGO/TiO_2_	solar simulator (AM1.5G, 100 mW cm^–2^)	H_2_O reduction (MeOH)	–	21500	–	–	14.3^c^	d	(193)
Au/CdSQDs/CeO_2_	150 W Xe lamp (λ > 400 nm)	H_2_O reduction (Na_2_SO_3_/Na_2_S)	–	12475^c^	>6	–	1.7	d	(194)
BP/Au nanorods/CdS nanowires	Xe lamp (λ ≥ 420 nm, 300 mW cm^–2^)	H_2_O reduction (Na_2_SO3/Na_2_S)	25	8600	>9	2.3 (@900 nm)^a^		–	(189)
Bi–Bi_2_MoO_6_/CdS-DETA	300 W Xe lamp (λ > 420 nm)	H_2_O reduction (Na_2_SO_3_/Na_2_S)	–	7370	>16	–	1.5^c^	–	(195)
Au/Pt/TiO_2_	300 W Xe lamp	H_2_O reduction (lactic acid)	–	6370^c^	–	–	∼3.2^c^	–	(196)
Au/Pt/Fe_2_O_3_/g-C_3_N_4_	150 W Xe lamp	H_2_O reduction (TEOA)	–	4730	>12	–	1.54^c^	–	(197)
AuNPs/TiO_2_ nanosheets	350 W Xe lamp	H_2_O reduction (glycerol)	–	4700	>1^d^	–	–	–	(198)
Au/rGO/Pt	visible light (λ > 420 nm)	H_2_O reduction (MeOH)	–	4333^c^	–	0.05^c^ (@1000 nm)	–	–	(180)
Au@Pt/ZIS	300 W Xe lamp (λ ≥ 420 nm)	H_2_O reduction (Na_2_SO_3_/Na_2_S)	25	4200	>20	6.23	10	–	(190)
Au/CuSe/Pt	300 W Xe lamp (λ > 420 nm)	H_2_O reduction (Na_2_SO_3_/Na_2_S)	–	4200^c^	>24	0.55 (@600 nm)	9.7	–	(191)
Cu/CG	300 W Xe lamp	H_2_O reduction (lactic acid)	–	3940	>0.1	–	–	–	(199)
AgPd/g-C_3_N_4_	300 W Xe lamp (λ ≥ 400 nm, 35 mW cm^–2^)	H_2_O reduction (TEOA)	–	3430	>28	8.43 (@420 nm)	b	–	(200)
Au/g-C_3_N_4_	300 W Xe lamp (λ > 400 nm)	H_2_O reduction (TEOA)	–	3308	–	–	348	–	(201)
GO-supported CdSe/CdS@Au	300 W Xe lamp	H_2_O reduction (Na_2_SO_3_/Na_2_S)	–	3113	>1	–	–	–	(192)
Au/WO_3_	300 W Xe lamp (λ > 420 nm)	H_2_O reduction (Na_2_SO_3_)	6	2450	>24	–	–	–	(202)
AuPd/g-C_3_N_4_	300 W Xe lamp (λ ≥ 400 nm, 35 mW cm^–2^)	H_2_O reduction (TEOA, K_2_HPO_4_)	4	2140^c^	>16	–	–	–	(203)
Au@Pt/g-C_3_N_4_	300 W Xe lamp (λ > 400 nm)	H_2_O reduction (TEOA)	–	1876	>6	–	3.6	–	(204)
Au_50_Pt_50_/C_3_N_4_	300 W Xe lamp	H_2_O reduction (TEOA)	25	1600	>10	–		3	(205)
Au/g-C_3_N_4_	solar simulator (150 W)	H_2_O reduction (TEA)	–	1300^c^	>3^a^	–	–	–	(206)
Pd^–^Ag/g-C_3_N_4_	direct sunlight (1100 ≥ λ ≥ 300 nm, 840 mW)	H_2_O reduction (TEA)	–	1250	>16	8.7	1.5	–	(207)

a Experiment duration.

b Neglible product detected without plasmonic material.

c Values extracted from graph or calculated.

d Neglible product detected without light.

**Table 8:** Metal–2D for H_2_ Production (2/2)

photocatalyst	light source	reaction	temp (°C)	formation rate (μmol g^–1^ h^–1^)	stability (h)	AQE (%)	PE	LE	ref
**PNP–2D for H**_**2**_									
Ag/TiO_2_/g-C_3_N_4_	300 W Xe lamp (λ > 420 nm)	H_2_O reduction (TEOA)	–	1120	>30	–	3.3	–	(208)
AgNPs/g-C_3_N_4_/SnS_2_	500 W Xe lamp (λ > 420 nm)	H_2_O reduction (Na_2_SO_3_/Na_2_S)	–	1105	–	–	1.8	–	(209)
Au/C_3_N_4_-MoS_2_	300 W Xe lamp (λ > 400 nm)	H_2_O reduction (TEOA)	6	1050^c^	>30	–	∼10^c^	–	(210)
PtAu/g-C_3_N_4_	300 W Xe lamp	H_2_O reduction (Na_2_SO_3_/Na_2_S)	43–45	1009	>20	–	10.7	–	(211)
Pd/C_3_N_4_	300 W Xe lamp (λ > 400 nm)	H_2_O reduction (TEOA)	5	758	>20	3.8 (@420 nm)	5	–	(212)
Ag NC/CNT/g-C_3_N_4_	300 W Xe lamp (λ > 400 nm)	H_2_O reduction (TEOA)	–	693	>20	–	91	–	(213)
Au/g-C_3_N_4_	300 W Xe lamp (λ > 400 nm)	H_2_O reduction (TEOA)	–	565	>20	–	∼3.7^c^	–	(214)
Au/g-C_3_N_4_	300 W Xe lamp (λ > 420 nm)	H_2_O reduction (TEOA)	20	540	>9^a^	0.1 (@520 nm)	54^c^	–	(182)
Au/P-doped g-C_3_N_4_	300 W Xe lamp	H_2_O reduction (MeOH)	–	460^c^	>30	∼3.6 (@420 nm)	∼12	–	(216)
Au/ZnO	solar simulator (AM1.5 filter, 300 W)	H_2_O reduction (Na_2_S/Na_2_SO_3_)	–	350.2	>12	–	b	–	(217)
Ag/2D white-C_3_N_4_	300 W Xe lamp (λ > 400 nm)	H_2_O reduction (hole scavenger)	–	342	>20	–	2.24	–	(218)
Au/g-C_3_N_4_	300 W Xe lamp (λ ≥ 400 nm)	H_2_O reduction (MeOH)	–	223	>5^a^	–	130	–	(219)
Au/g-C_3_N_4_	300 W Xe lamp (λ > 400 nm)	H_2_O reduction (TEOA)	25	146.2	>3	–	5.3	–	(220)
Ag@MoS_2_/g-C_3_N_4_	300 W Xe lamp (λ > 420 nm, 173 mW cm^–2^)	H_2_O reduction (TEOA)	6	104^c^	>20	–	2.08	–	(221)
Au/Ni_2_P/g-C_3_N_4_	300 W Xe lamp (λ > 400 nm)	H_2_O reduction (TEOA)	–	78.65	>22	0.04 (@420 nm)	1.67	–	(222)
Au/g-C_3_N_4_/In_2_O_3_	150 W Xe lamp (λ > 420 nm)	H_2_O reduction (MeOH)	–	56.5	>28	2.5 (@420 nm)	1.93^c^	–	(223)
Au nanorods/MoS_2_	100 W Xe lamp (λ > 460 nm)	H_2_O reduction (lactic acid)	25 (RT)	18.67^c^	>9	0.2 (@780 nm)	b	–	(181)
Bi/WN	300 W Xe lamp (λ > 700 nm)	H_2_O reduction (TEA)	4	7.5	>20	0.16 (NIR)	2.47	–	(224)
Au_1_Pd_2_/GO	500 mW cm^–2^	FA dehydrogenation	25	15.9 TOF (min^–1^)	–	–	–	1.09	(225)

a Experiment duration.

b Negligible product detected without plasmonic material.

c Values extracted from graph or calculated.

**Table 9:** Plasmonic Metal–2D for CO_2_ Reduction

photocatalyst	light source	major product	temp (°C)	formation rate (μmol g^–1^ h^–1^)	minor products	selectivity (%)	stability (h)	QE (%)	PE	LE	ref
**PNP–2D for CO**_**2**_											
Au/ZIS/g-C_3_N_4_	300 W Xe lamp	CO	10	242.3	CH_4_, H_2_	94.1	>5^a^	–	–	d	(230)
Ag nanocube/rGO	300 W Xe lamp	CO	45	133.1	–	∼100	>6^a^	–	3.1^c^	–	(184)
Ag nanosphere/rGO	300 W Xe lamp	CO	45	120.1	–	∼100	>6^a^	–	2.8^c^	–	(184)
Ag/MoS_2_	300 W Xe lamp (150 mW cm^–2^)	CO	15	74.7^c^	H_2_	98	>6^a^	–	–	–	(74)
Au/g-C_3_N_4_	Xe lamp	CO	–	28.3	CH_4_	85	–	–	7.6	–	(231)
Au/g-C_3_N_4_	Hg lamp (8 W)	CO	–	9.7^c^	CH_4_	–	>8^a^	–	6	–	(185)
Au/g-C_3_N_4_	300 W Xe lamp (λ > 420 nm)	CO	–	6.59	CH_4_	80.9	>2^a^	–	–	–	(232)
AgPd/g-C_3_N_4_	solar simulator (AM1.5G, 100 mW cm^–2^)	CO	–	5.42	–	–	>20	–	c	–	(227)
Au/P-doped g-C_3_N_4_	300 W Xe lamp	CH_4_	–	120^c^	H_2_	–	>30	2.83^e^ (@420 nm)	6	–	(216)
AgPd/N-doped TiO_2_	300 W Xe lamp	CH_4_	25	79.0	–	∼100	>50	–	b	–	(233)
Au/TiO_2_	300 W Xe lamp	CH_4_	–	70.34	CO	80	>100 s^a^	–	∼70^c^	–	(234)
Au/MoS_2_	300 W Xe lamp (150 mW cm^–2^)	CH_4_	15	19.38^c^	H_2_, CO	80.2	>6^a^	–	–	–	(74)

a Experiment duration.

b Negligible product detected without plasmonic material.

c Values extracted from graph or calculated.

d Negligible product detected without light.

e Definition not reported.

### Plasmonic Metal–2D Hybrids for Hydrogen Production

By tuning the shape and size of the metal nanoparticles, the solar light absorption profiles of the hybrid system can be modified to ensure the optimum synergy in between metal and 2D materials.^180−184^ To study the effect of the metal nanoparticle shape, Zhang et al. compared the photocatalytic H_2_ generation performances of 2D Molybdenum disulfide (MoS_2_) decorated with Au nanospheres (AuNSs), nanorods (AuNRs), and triangles (AuNTs).^181^ To eliminate the band gap excitation of MoS_2_, visible light was used, and negligible amounts of H_2_ were detected using either pristine Au nanostructures or MoS_2_. However, as shown in Figure 5c, all three hybrid structures were able to catalyze H_2_ generation. Among the three types of Au nanostructures, AuNR-MoS_2_ exhibited the best performance. The H_2_ generation rate of AuNR/MoS_2_ was 1.6 and 3.7 times higher than those of AuNT/MoS_2_ and AuNS/MoS_2_. This superior hydrogen generation of AuNR-MoS_2_ suggests that efficient plasmon-induced hot-electron transfer at the metal–2D interface is promoted by the stronger electric field from AuNRs (see Figure 5b). The authors also found that the slower recombination of electrons and holes in AuNRs contributed to charge separation in both AuNR and MoS_2_ across the Schottky barrier.

The mass ratio between the metal particles and the 2D materials is another determining factor of the photocatalytic reaction kinetics.^182,212,218,220^ Guo et al. examined the role of the metal particle loading amount. They loaded 18 nm AuNPs with varying mass percentages ranging from 0.5 to 4.0% onto graphitic carbon nitride (g-C_3_N_4_) nanosheets and investigated them toward photocatalytic H_2_ generation. As can be seen from Figure 5d, the H_2_ evolution rate increased until the loading amount reached an optimum and then decreased with continuing increase of the mass ratio of AuNPs. The number of the particles per area increases as the loading amount increases. After a certain loading amount, the close vicinity of the metal particles promotes electron–hole recombinations.^226^ This finding also implies that the optimum loading mass increases with increased size of the metal nanoparticles. This is because for larger particles to achieve the same number density, the required total mass of the loaded particles will also increase. The effect of metal nanoparticle size is described in more detail in the CO_2_ reduction section.

Co-loading or alloying metal–metal nanoparticles can further influence the photocatalytic activity of the hybrid system.^187,200,203,207,227^ Cheng et al. demonstrated that the H_2_ generation could be benefited from co-loading Au clusters and AuNPs onto g-C_3_N_4_.^228^ Au cluster here refers to an “ultrasmall nanoparticle” that consists of tens to hundreds of gold atoms and has quantized energy levels. The authors argued that this configuration allows Au clusters to act as electron acceptors to reduce the charge recombination within AuNPs; hence, increasing the hot-electron lifetime within the particles. The H_2_ generation rate of the AuNP cluster-NP/g-C_3_N_4_ increased by a factor of 6 compared to AuNP/g-C_3_N_4_ and by 20 times compared to Au cluster/g-C_3_N_4_. This was attributed to a combined effect of a lowered Schottky barrier between Au and g-C_3_N_4_ via strong sp^2^ hybridization and the prolonged hot-carrier lifetime. This enhancement mechanism also applies to bimetallic system such as AuPd loaded g-C_3_N_4_.^229^ For instance, Han et al. demonstrated that by alloying Au and Pd, the H_2_ production rate of the alloy–g-C_3_N_4_ hybrid nanocomposite was increased by 3.5 and 1.1 times as compared to Au–g-C_3_N_4_ and Pd–g-C_3_N_4_, respectively. Their investigations on the nanocomposites indicated that the lifetime of the photogenerated hot carriers in the AuPd alloy was longer than those in Au and Pd and therefore inhibited the recombination of the hot carriers and facilitated greater photocatalytic H_2_ generation.

Due to the high cost of noble metals, researchers also started to explore non-noble plasmonic metals.^186,212^ In Tables 7 and 8, less than 5 out of more than 30 hybrid systems studied toward H_2_ production are non-noble-metal-based. Xu et al. demonstrated that Cu as a non-noble metal is also capable of enhancing photocatalytic water-splitting when combined with 2D tungsten disulfide (WS_2_).^186^ Their Cu-WS_2_ heterostructure improved the H_2_ generation rate by 40- and 2.2-fold as compared to the WS_2_ and Cu single-component system under simulated 1 sun irradiation (Figure 5f). This study not only showed the potential of non-noble metals as a proof of principle, it also reached one of the highest H_2_ production rates (64 000 μmol g^–1^ h^–1^) and exhibited one of the highest plasmonic activity enhancements (40) in Table 9.

To conclude, upon combining metal nanostructures and 2D materials, the hybrid systems exhibited improved HER performances. Plasmonic materials, due to their superior light absorption and unique optical properties, can enhance the light-harvesting of 2D materials and extend their usable wavelength range. Through proper engineering of plasmonic metal–2D hybrid systems, the recombination rate of the photogenerated carriers can also be reduced significantly, which results in improved H_2_ production.

### Plasmonic Metal–2D Hybrids for CO_2_ Reduction

In the H_2_ generation section, it was briefly mentioned that the size of the metal nanoparticles can also influence the photocatalytic activity of plasmonic metal–2D hybrid systems. To examine this, Li et al. grew AuNPs of various sizes on g-C_3_N_4_ nanosheets for CO_2_ reduction.^183^ They showed that AuNPs with 4–6 nm diameter exhibited the best CO_2_ reduction performance under visible light irradiation (Figure 5e). The Fermi level of the smaller particles is lower. This means the Schottky barrier between AuNP and g-C_3_N_4_ is smaller and the photogenerated electrons are more readily transferred from g-C_3_N_4_ to AuNPs.

As concluded in multiple of the aforementioned studies, the band structure at the Schottky junction is crucial to the photocatalytic activity of the system. A material-dependent study by Sun et al. comparing the CO_2_ photoreduction performance of Au–MoS_2_ and Ag–MoS_2_ further demonstrated the role of the band structures at the heterojunctions.^74^ This study showed that the band structure at the metal–2D interface can impact the formation rates of specific reduction products and their selectivity (Figure 5g). At the Au–MoS_2_ interface the band structure prevented the electron transfer from MoS_2_ to Au, whereas the hot-electrons from Au could inject to MoS_2_. This resulted in electron accumulation on MoS_2_ upon light irradiation. On the other hand, at the Ag–MoS_2_ junction, the electron transfer from MoS_2_ to Ag was favored resulting in less electrons being accumulated at MoS_2_. Subsequently, in the Ag-based system, mainly CO was generated, whereas in the Au-based system CH_4_ was the major product. This result shows that the selectivity in CO_2_ reduction processes can be carefully controlled by tuning the composition of plasmonic metal–2D nanocomposites.

Optimization of the Schottky junction at metal–2D interfaces can prolong the hot carrier lifetime and improve the charge transfer to promote the photocatalytic performance of the hybrid systems. Moreover, particularly for CO_2_ photoreduction, the band structure at the interface plays a significant role in selectivity. This provides us with immense opportunities in photocatalytic system development.

Even though the plasmonic metal–2D hybrid systems have exhibited excellent potential for synergetic photocatalytic performance, the large-scale reproducibility of conventional 2D materials still remains as a bottleneck. Both “top-down” exfoliation and the “bottom-up” synthesis are not easily scalable yet. Although tremendous efforts have been devoted to the mass production of 2D materials, there remain substantial challenges before they can make a real impact in industrial applications.^235^ Chemically synthesized nanosheets and nanoplates have been gaining popularity as alternative 2D materials due to their producibility and photocatalytic performance. Therefore, chemically synthesized 2D materials with good crystallinity and dispersity and their plasmonic hybrid systems deserve further exploration for photocatalytic applications.

## Plasmonic Metal–MOF

**Figure 6:**
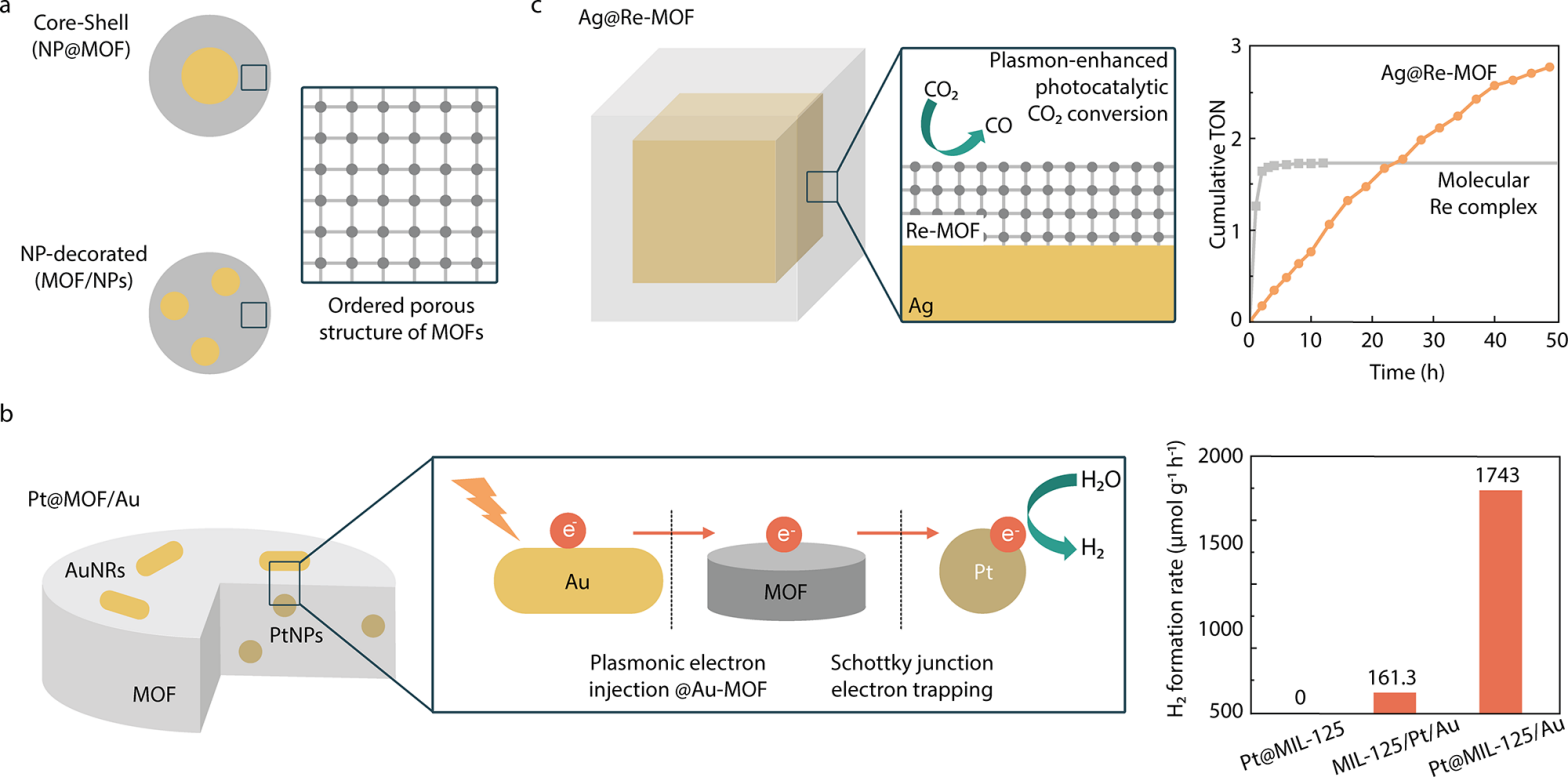
Plasmonic metal–MOF hybrids. (a) Types of plasmonic metal–MOF hybrids. (b) Structure of Pt@MOF/Au and scheme of the charge transfer that enhances the catalytic activity toward water-splitting; H_2_ formation rates of different modifications of the catalyst. (c) Left: Structure of Ag@Re-MOF and scheme of the spatial confinement of the catalytic Re-MOF to the surface of the plasmonic silver; right: cumulative TON of the catalytic Re complex incorporated into the hybrid compared to the TON of the molecular Re catalyst. (b) Adapted with permission from ref. (89). Copyright 2017 Elsevier. (c) Adapted with permission from ref. (85). Copyright 2017 American Chemical Society.

Combining plasmonic light-harvesting of PNPs with the property tool set that MOFs offer allows for a vast amount of synergistic possibilities. In these hybrids, MOFs and PNPs can be structurally combined in several ways. Two of the most prominent examples are MOFs grown around PNPs and MOFs that are decorated with PNPs (see Figure 6a).^81,82,236^ In the following, a unified nomenclature is used to increase readability. Particles assembled on other compounds are positioned after a “/”-sign while incorporated components are written before an “@”-sign. From left to right, the components are arranged from core to shell. Furthermore, only the common names and important features of the respective MOFs are mentioned in the text as another measure to increase readability. For example, MIL-125, a framework built up from 1,4-benzenedicarboxylate (organic linker) and TiO_2_ (metal node), will be described as a semiconductor-like MOF.

### Plasmonic Metal–MOF Hybrids for H_2_ Production

Recently explored hybrids that exploit the combination of PNPs and MOFs to boost the performance of photocatalytic H_2_ production are summarized in Table 10. One of the highest H_2_ production rates was reported for Pt@MIL-125/Au. The hybrid catalyzed the water-splitting reaction with a H_2_ formation rate of 1743 μmol g^–1^ h^–1^.^89^ In the optimized hybrid, PtNPs were incorporated into a porous semiconductor-like MOF (MIL-125), and the structure was subsequently decorated with rod-shaped AuNPs (see Figure 6b). The porosity of the MOF allows the reactants and products to access and leave the catalytic sites, respectively. Under visible light radiation, hot electrons generated at the AuNPs were steered through the lowest unoccupied molecular orbital (LUMO) of the MOF toward the PtNPs. Due to the difference in work functions of the MOF (semiconductor-like) and the Pt (metal), electrons left the MOF in favor of the Pt surface and were trapped there (Schottky junction; see Figure 6b). This electron transport pathway resulted in an accelerated unidirectional charge transfer and an amplified e^–^–h^+^ separation. The extent of e^–^–h^+^ separation was greatly influenced by the spatial separation of AuNPs and PtNPs. Figure 6b shows the impact of incorporating the PtNPs into the MOF compared to assembling both the PtNPs and the AuNPs on the MOF surface (MIL-125/Pt/Au), where the formation rate is about 10 times lower. Figure 6b also illustrates the role of the plasmonic component in harvesting the light as no catalytic activity was reported without the AuNPs (0 μmol g^–1^ h^–1^ for Pt@MIL-125).

**Table 10:** Plasmonic Metal–MOF Hybrid Systems for H_2_ Generation and CO_2_ Reduction

photocatalyst	light source	reaction	temp (°C)	formation rate (μmol g^–1^ h^–1^)	stability (h)	AQE (%)	PE	LE	ref
**PNP–MOF for H**_**2**_									
MIL-101/Au@CdS	300 W Xe lamp (λ > 420 nm)	H_2_O reduction (Na_2_S/Na_2_SO_3_)	–	25000	>8	8.8 (@420 nm)	1.3^b^	c	(237)
UiO-66(Zr_85_Ti_15_)/Au@Pd	500 W Xe lamp (λ > 420 nm, 320 mW cm^–2^)	FA dehydrogenation	25 (RT)	3315^b^	>6	–	12.6^b^	1.5	(86)
Pt@MIL-125/Au	300 W Xe lamp (800 > λ > 380 nm)	H_2_O reduction (TEOA)	25 (RT)	1743	>6	–	a	–	(89)
MIL-101@anatase/Au	400 W tungsten lamp (λ > 400 nm)	H_2_O reduction (MeOH)	17	903	>6	–	a	–	(238)
UCNPs/Pt@UiO-66-NH_2_/Au	solar simulator (AM1.5G, 300 W)	H_2_O reduction (TEOA)	25 (RT)	280	>16	–	∼3	–	(239)


photocatalyst	light source	major product	temp (°C)	formation rate (μmol g^–1^ h^–1^)	minor products	selectivity (%)	stability (h)	AQE (%)	PE	LE	ref
**PNP–MOF for CO**_**2**_											
PPF-3/Au	300 W Xe lamp (λ > 400 nm)	formic acid	25 (RT)	42.7	–	100	>24	–	5	–	(240)
MIL-101(Cr)/Ag	300 W Xe lamp (780 > λ > 400 nm, 200 mW cm^–2^)	CO	50	808.2	CH_4_, H_2_	∼61^a^	>18	–	27^a^	–	(241)
Au@Pd@MOF-74	500 W Xe lamp	CO	–	2.46^b^	–	100	>5	–	1.7^a^	c	(242)
Ag@Re_3_-MOF	300 W Xe lamp (700 > λ > 400 nm)	CO	–	0.94 TON/h^b^	–	∼100	>48	–	7	–	(85)
Au@Pd@MOF-74/Pt	500 W Xe lamp	CH_4_	–	2.47^b^	CO	∼84	>5	–	1.4^a^	c	(242)
ZIF-67/Au	solar simulator (150 mW cm^–2^)	MeOH	–	2380^b^	EtOH	∼97^b^	>12	6.4 (@420 nm)	a	–	(87)

a Negligible product detected without plasmonic material.

b Values extracted from graph or calculated.

c Negligible product detected without light.

Like the previous hybrid, UiO-66(Zr_85_Ti_15_)/Au@Pd was designed to enrich the surface of the catalytic compound (here Pd) with electrons.^86^ Here, a MOF (UiO-66(Zr_85_Ti_15_)) with photoactive linkers and doped metal nodes was decorated with plasmonic core–shell Au@Pd nanoparticles resulting in the second highest formation rate reported (see Table 10). This combination resulted in several processes upon visible light radiation that boosted the hydrogen formation from formic acid. First, doping the metal nodes (Zr) of the MOF with Ti promoted the charge transfer of the electrons created in the MOF linkers and enhanced the visible light absorption of the MOF. Second, amine groups within MOFs enhance the catalytic activity as they assist in the dissociation of O–H as an important step for the transformation of formic acid to H_2_. And last, hot electrons created by the LSPR in the Au and photoexcited electrons created at the photoactive MOF-linkers are transferred to the catalytic Pd surface. The plasmonic component is essential for the catalytic performance, as the hybrid has a 12.6 times higher production rate with Au compared to without. The resulting formation rate of 3315 μmol g^–1^ h^–1^ is the second highest reported in Table 10. More importantly, the hybrid shows how the building blocks of MOFs can be smartly designed to, for example, promote certain steps in the synthesis, facilitate charge transfer, or enhance absorption properties in combination with PNPs.

While the concepts used in the previously discussed hybrids are promising, by far the highest formation rate with 25 000 μmol g^–1^ h^–1^ was reported for MIL-101/Au@CdS catalyzing the water-splitting reaction.^237^ Here, Au@CdS core–shell particles were assembled on a MOF structure (MIL-101). The hybrid exploited the already highly reactive catalyst CdS (7400 μmol g^–1^ h^–1^; semiconductor) for achieving high formation rates. By assembling CdS on the MOF-surface, the formation rate was enhanced 2.7-fold. The MOF acts as a support, resulting in well-dispersed CdS particles and therefore possibly more active catalytic sites. By absorbing visible light, the MOF generated excited electrons that enriched the surface of CdS, therefore facilitating the reaction. Introducing AuNPs in the form of core–shell Au@CdS particles enhanced the reactivity by another 30%. The authors propose that the AuNPs accelerated the charge transfer and additionally extended the response spectrum of CdS by the LSPR.

When comparing the values in Table 10, two approaches for H_2_ production utilizing PNP–MOF hybrids stand out. In absolute numbers, introducing an additional material that is already highly catalytically active (e.g., the semiconductor CdS)^237^ that can be further enhanced by PNPs and MOFs can result in very high formation rates. In relative numbers (enhancement), targeting single, crucial steps in reactions by introducing functional groups to the MOF seems especially promising.^86^ In all systems reported for H_2_ production, the hybrid consisted of three components, with the PNPs assembled on the MOF-surface (see Table 10). Different, so far unexplored geometries and structural assemblies could result in new features that improve the catalytic performance.

### Plasmonic Metal–MOF Hybrids for CO_2_ Production

One of the most recognized works and one of the first PNP–MOF hybrids used as photocatalyst for CO_2_ conversion was Ag@Re_3_-MOF (see Figure 6c).^85^ Here, a molecular CO_2_-to-CO conversion photocatalyst (Re^I^(CO)^3^(BPYDC)Cl) was covalently incorporated into a MOF (UiO-67) which was grown around cubic AgNPs. This combination yielded two main advantages. On the one hand, the incorporation of the molecular compound into the rigid MOF structure prevented its dimerization and increased the stability of the photocatalyst 48-fold to 48 h. On the other hand, coating the AgNPs with the MOF spatially confined the catalytic Re centers close to the metal surface. Under visible light illumination, the AgNPs created an intensified near-field that could be absorbed by the incorporated Re photocatalyst. As a result, the CO_2_-to-CO conversion was enhanced 7-fold due to the incorporation of the PNP.

Another interesting system worth mentioning, although not in the liquid phase, was studied by Halas et al.^88^ For this, they combined aluminum nanocubes (AlNCs) with a MOF known for a high CO_2_ gas uptake. The hybrid system was used to catalyze the reverse water gas shift reaction (CO_2_ + H_2_ → CO + H_2_O) in the gaseous phase. The hybrid exhibited a ∼4.5-fold increased CO_2_ uptake compared to the pristine AlNCs. The CO_2_ preconcentration near the surface of the plasmonic material resulted in a ∼3-fold increase of CO production compared to the pristine AlNCs.

By far the highest product formation rate for CO_2_ conversion in Table 10 was reported for a hybrid containing a zeolitic imidazolate framework (ZIF), a subclass of MOFs. This subclass of MOFs has promising features for CO_2_ reduction and is currently explored for CO_2_ conversion using various approaches and materials.^243−245^ Two of the main limiting factors of current ZIF materials are sufficient solar light absorption and e^–^–h^+^ separation.^246^ Combining ZIFs with PNPs may be a perfect synergistic fit, as PNPs can bring both of these features to the system. In the reported hybrid, ZIF-67 decorated with AuNPs generated methanol (MeOH) with a rate of 2380 μmol g^–1^ h^–1^ and a selectivity of 97%, while also being stable for at least 12 h.^87^ It was proposed that hot electrons generated in the AuNP upon illumination could overcome the Schottky barrier formed at the PNP–MOF interface. As a result of the electrons being irreversibly injected into the LUMO of the MOF, the lifetime of the carriers was extended resulting in an electron-enriched MOF surface where the reaction subsequently occurs. Both components of the hybrid were crucial for the catalytic performance. While the MOF provided the catalytic sites, no product was detected without the plasmonic material.

In the majority of the reported hybrids, the PNP loading amount (wt%) was optimized. However, the size of the MOF crystals was not. Guo et al. showed for MIL-101(Cr)/Ag that controlling the MOF size can have a high impact on the catalytic performance of a PNP–MOF hybrid.^241^ When reducing the MOF crystal size in the hybrid from 800 to 80 nm, the CO_2_-to-CO conversion rate was increased ∼20-fold. The authors attributed this to the increased fraction of highly catalytic edge and corner sites of the MOF. This shows that all components should be carefully tuned to optimize the catalytic performance.

The examples for both H_2_ production as well as CO_2_ reduction show that PNP–MOF hybrids have various potential pathways to increase activity and selectivity. Interestingly, the highest formation rates for both H_2_ production as well as CO_2_ reduction were achieved by NP-decorated hybrids. For the further development of PNP–MOFs, hybrids could be designed that facilitate single, crucial steps in reactions by introducing functional groups to the MOF.^86^ This could open energetic bottlenecks of reactions and show new paths for catalysis. Furthermore, both the spatial arrangement of the hybrid components^89^ and the size of the MOF crystals^241^ could be implemented as common screening parameters. Generally, data concerning the long-term stability of MOF-PNP hybrids is currently not available, and only two hybrids have been reported with a stability of at least 24 h (see Table 10). This shows that long-term stability is an issue to be addressed for potential industrial applications.

## Plasmon-Assisted Electrocatalysis

Despite the great progress in photocatalysis, the solar-to-fuel conversion efficiency is still low. Compared to plasmonic photocatalysis, electrocatalysis shows a much higher hydrogen generation and CO_2_ reduction activity. However, the high overpotential and low current density are the current bottlenecks hindering energy efficient industrial applications. Consequently, combining plasmonics with electrocatalysis is a promising strategy to improve the energy conversion efficiency.

### Plasmon-Assisted Electrochemistry for H_2_ Production

All different types of hybrid systems that were discussed above for photocatalytic H_2_ generation may also be useful for plasmon-enhanced electrocatalytic applications. Therefore, the broad spectrum of materials investigated is summarized in Table 11. It shows plasmonic metal and semiconductor, 2D and MOF hybrid systems. In this table, the observables *Overpotential* and *Tafel slope* are given both under illumination and in dark (the values for dark conditions are given in brackets). The comparison of values in dark and under illumination allows for an evaluation of the enhancement that plasmonics provides to the hybrid system. This evaluation can only be qualitative as the changing observables may not exclusively be attributed to the plasmonic enhancement.^248^ Both in the table and in the discussion below, all overpotentials are reported at a current density of 10 mA cm^–2^.

**Table 11:** Plasmonic Electrocatalysts for HER H_2_ Generation (1/2)

			operation - irradiated (dark)		
catalyst	light source	electrolyte	overpotential @ 10 mA cm^–2^ (mV vs RHE)	Tafel slope (mV dec^–1^)	ref
**PNP–Metal for H**_**2**_					
Pt/Fe@ Au nanorods	laser (@808 nm)	0.5 M H_2_SO_4_	18 (43)	29.3 (30.6)	(215)
PtNiCu	300 W Xe lamp (780 > λ > 420 nm)	0.5 M H_2_SO_4_	39 (51)	24.1 (27.3)	(249)
Au nanorods@ PdAg nanosheets	300 W Xe lamp	0.5 M H_2_SO_4_	50 (80)	40.5 (59.7)	(250)
AgPt-tipped Au nanostars	300 W Xe lamp (1100 > λ > 420 nm)	0.5 M H_2_SO_4_	58 (75)	35 (37)	(247)
AgPt-edged Au nanostars	300 W Xe lamp (1100 > λ > 420 nm)	0.5 M H_2_SO_4_	85 (87)	48 (49)	(247)
Pd-tipped Au nanorods	300 W Xe lamp (1100 > λ > 420 nm)	0.5 M H_2_SO_4_	87 (167)	105 (129)	(251)
AgPt-covered Au nanostars	300 W Xe lamp (1100 > λ > 420 nm)	0.5 M H_2_SO_4_	91 (119)	53 (60)	(247)
Ag/Ag_2_S/NiS_2_	LED (780 > λ > 380 nm)	1 M KOH	95 (151)	45 (74)	(252)
Cu_2–*x*_@Au_2_S@Au nanoplates	laser (@532 nm, 1051 mW cm^–2^)	0.5 M H_2_SO_4_	96 (152)	– (118)	(253)
PtPd-decorated Au@Ag nanocrystal	300 W Xe lamp (1100 > λ > 420 nm)	0.5 M H_2_SO_4_	150 (199)	101 (116)	(254)
AuNPs@rGO@Pd	300 W Xe lamp (λ > 420 nm)	0.1 M KOH	291 (342)	86 (162)	(255)
Ag/Au HPNS@ porous Fe_2_P	300 W Xe lamp (20 mW cm^–2^)	0.5 M H_2_SO_4_	350 (402^a^)	97 (−)	(256)

**PNP–Semiconductor**					
Au film@Ti/TiO_2_	laser (@980 nm, 2 W cm^–2^)	0.5 M H_2_SO_4_	45 (183)	35 (119)	(257)
Au@NiCo LDH	solar simulator (AM 1.5G, 100 mW cm^–2^)	1 M KOH	119 (160)	57.5 (62.1)	(258)
AuNPs@ Bi_2_Se_3_ nanoflowers	iodine tungsten lamp	0.5 M H_2_SO_4_	375 (380)	78 (82)	(296)
**PNP–2D for H**_**2**_					
AuNPs/MoS_2_/graphene/Ni foam	solar simulator	0.5 M H_2_SO_4_	28 (41)	26 (36)	(259)
1T-phase Au/Pd-MoS_2_ nanosheets	Xe lamp (300 mW cm^–2^)	0.5 M H_2_SO_4_	64^a^ (119^a^)	49 (63)	(260)
Au@ N-doped carbon	532 nm laser, 1 W	0.5 M H_2_SO_4_	99 (196)	77 (87)	(261)
Cu_1.75_S-Au@ MoS_2_ monolayer	laser (@650 nm, 1 W cm^–2^)	0.5 M H_2_SO_4_	114.5 (224.3)	39 (47)	(262)
Ti_3_C_2_T_*x*_ MXene	laser (@808 nm, 7.17 W cm^–2^)	0.5 M H_2_SO_4_	128 (578)	91 (160)	(93)
MoS_2_ monolayer@nanoporous Au	Xe lamp (1 W cm^–2^)	0.5 M H_2_SO_4_	149 (167)	38 (49)	(297)
Au nanorods@MoS_2_ nanosheets	laser (@808 nm, 1.5 mW)	0.5 M H_2_SO_4_	160 (220)	71 (86)	(263)
MoS_2_@Au	500 W lamp (1100 > λ > 400 nm)	0.5 M H_2_SO_4_	193^a^ (316^a^)	74 (93)	(264)
Au nanorods@MoS_2_ nanosheets	laser (@721 nm, 1 W cm^–2^)	0.5 M H_2_SO_4_	220 (260)	63 (82)	(265)
Au@Ag nanorattles@MoS_2_ monolayer	laser (@690 nm, 5 mW)	0.5 M H_2_SO_4_	335^a^ (448^a^)	155 (175)	(266)
Ag/TiO_2_/RGO	visible light (1.22 × 10^5^ lx)	0.1 M KOH	820 (920)	– (165)	(267)

**PNP–MOF**					
Au nanorods@CoFe-MOF NS	laser (@808 nm, 1500 mW cm^–2^)	0.5 M H_2_SO_4_	292^a^ (435^a^)	94 (115)	(298)
Au nanorods@Co-MOF NS	laser (@808 nm, 200 mW cm^–2^)	0.5 M H_2_SO_4_	540 (690)	133 (175)	(268)

a Values extracted from graph or calculated.

**Figure 7:**
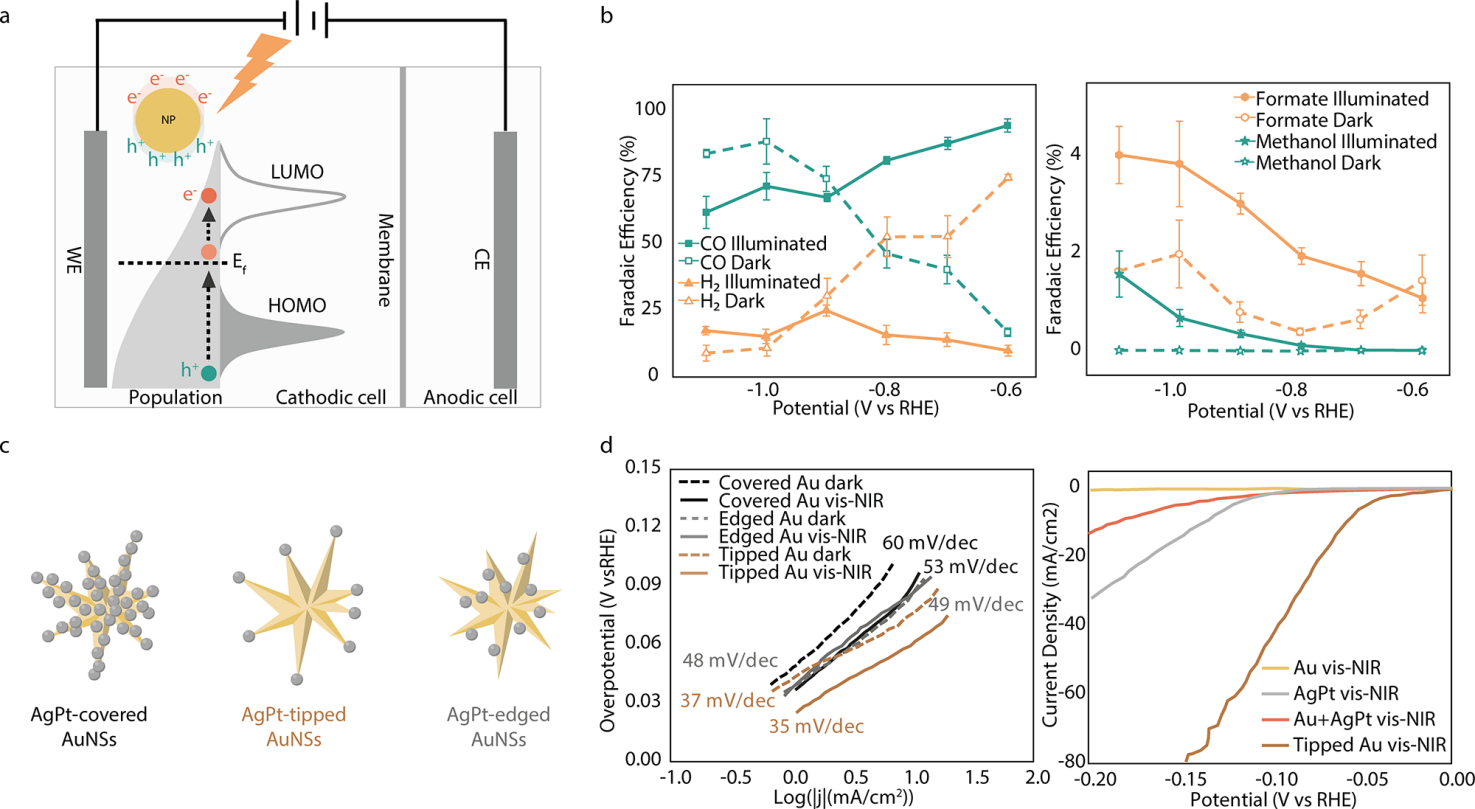
Plasmon-assisted electrocatalysis. (a) Schematic illustration of the experimental setup and mechanism for plasmon-assisted electrocatalysis. (b) By plasmon excitation, the selectivity toward desired products of the CO_2_RR can be increased. (c) Schematic illustration of AgPt-covered, -tipped, and -edged Au nanostars. (d) Corresponding behavior in Tafel slope and Overpotential in dark and under illumination for configurations in (c) and control samples. (a) Adapted with permission from ref. (92). Copyright 2020 American Chemical Society. (b) Adapted with permission from ref. (94). Copyright 2019 American Chemical Society. (c,d) Adapted with permission from ref. (247). Copyright 2019 Elsevier.

A study conducted by Wei et al. shows the effect different configurations of the same metal–metal hybrids can have on the performance.^247^ They probed the plasmon-enhanced electrocatalytic activity of Au nanostars modified with AgPt alloys in three different arrangements: AgPt-covered, -tipped, and -edged Au nanostars (see Figure 7c). The Au nanostars offer a wide range of tunability. By tuning the size of both the nanostar core and the nanostar branches, different resonant wavelengths can be achieved. Additionally, the position of the AgPt NPs allows further tuning, as, for example, the electromagnetic field has its highest enhancement in so-called “hot spots” at the nanostar tips.^269^ All three systems showed reduced overpotentials and Tafel slopes under visible–NIR irradiation. However, when comparing the configurations, the AgPt-tipped Au nanostars showed a superior performance with an overpotential of 58 mV_RHE_. This was 17 mV lower than for the AgPt-edged and 33 mV lower than for the AgPt-covered Au nanostars (see Figure 7d). Together with the reduced Tafel slope of the AgPt-tipped Au nanostars (35 mV/dec) this implied a more efficient charge transfer and faster reaction kinetics of this modified nanostar type. The superior activity was reported to be a result of the best synergy between the Au nanostars acting as light absorbers, thereby generating heat and exciting electrons, and the AgPt alloys as electron acceptors that provided the reactive sites.

Another interesting example highlights the synergistic effect that can arise between PNPs and conventional semiconductor electrocatalysts for solar-light-enhanced water-splitting. Du et al. investigated a catalyst consisting of AuNPs and NiCo layered double hydroxides (Au@NiCo LDH) toward both reactions involved in water-splitting, the HER and the oxygen evolution reaction (OER).^258^ To reach a current density of 10 mA cm^–2^, the Au@NiCo LDH required an overpotential of 119 mV for the HER and 203 mV for the OER under illumination. The comparison with the overpotentials needed in dark (160 mV for HER and 234 mV for OER) suggested that the plasmonic excitation of Au led to a higher charge transport efficiency. This shows the potential of incorporating plasmonic materials into conventional electrochemical systems to significantly reduce the electrical energy consumption.

Seven out of 11 metal–2D hybrids in Table 11 contain MoS_2_. While multiple configurations of this 2D material with different plasmonic metals have been explored,^260,262,263,265,297^ to our knowledge, one of the first metal–MoS_2_ hybrids for plasmon-enhanced electrocatalytic H_2_ generation was reported by Shi et al. in 2015.^263^ It consisted of Au nanorods (AuNRs) and MoS_2_ nanosheets. When illuminated with a 808 nm laser, matching the plasmon resonance of the AuNRs, the onset potential decreased by 60 mV compared to the dark condition. Under the same laser illumination, the activation energy of H_2_ evolution on the AuNR-MoS_2_ was calculated to be 75.65 kJ mol^–1^, much lower than that of MoS_2_ alone (94.82 kJ mol^–1^). Moreover, the plasmon excitation resulted in the Tafel slope of AuNR-MoS_2_ decreasing by 15 mV/dec. This indicates a higher hydrogen coverage on the surface of the catalysts and shows that plasmon excitation can enhance the electrochemical hydrogen evolution not only thermodynamically but also kinetically.

To our knowledge, so far only two PNP–MOF hybrid systems^268,298^ have been investigated toward plasmon-assisted electrochemical H_2_ generation. Currently, PNP–MOF systems seem to be significantly weaker in performance than the majority of the metal, semiconductor, and 2D hybrids, with the lowest overpotential reported thus far being 292 mV. In comparison, the best PNP–metal,^215^ PNP–semiconductor,^257^ and PNP–2D^250^ hybrids operated at overpotentials as low as 18, 45, and 28 mV. To conclude, plasmon excitation can improve the electrocatalytic hydrogen evolution activity both thermodynamically and kinetically. With Au being the plasmonic component in 20 out of the 23 listed configurations, it is still the most utilized plasmonic material in all plasmonic electrocatalysts for H_2_ generation reported in Table 11. One emerging non-noble plasmonic material is titanium nitride (TiN) which has shown superior performance for photoelectrochemical water-splitting.^60^ The remarkable hot carrier collection of TiN@TiO_2_ was attributed to its broadband absorption as well as the forming of an ohmic junction for efficient electron collection.^60^

### Plasmon-Assisted Electrochemistry for CO_2_ Reduction

Similar to electrochemical H_2_ production, the electrochemical reduction of carbon dioxide typically suffers from low reaction rates that necessitate high overpotentials. Additionally, the low selectivity, especially toward multicarbon products, impedes the electrochemical production of carbon-based fuels. Recent efforts to overcome these limitations by plasmon-assisted electrochemical (CO_2_) reduction are summarized in Table 12. Figure 7a shows the schematic setup and mechanism for plasmon-assisted electrocatalytic CO_2_ reduction. A study conducted by Kim et al. showed that plasmonic excitation can be used to increase the reaction rate of CO_2_ reduction.^271^ In this study, the electrodes consisted of Ag nanopyramids patterned on a Ag surface. The dark current density was −0.8 mA cm^–2^ under −1.1 V_RHE_. Under illumination the current density increased by 31%, up to −1.0 mA cm^–2^. The observed photocurrent was attributed to a resonant transfer of photogenerated plasmonic hot electrons to the lowest unoccupied molecular orbital (LUMO) acceptor energy levels of adsorbed CO_2_ molecules or reductive intermediates. This shows the potential of plasmon-assisted electrochemistry to overcome kinetic barriers.

**Table 12:** Plasmonic Electrocatalysts for CO_2_ Reduction

			operation point under illumination					
catalyst	light source	electrolyte	product	potential	current density (CD)	FE	LE	ref
**PNP–Metal for CO**_**2**_								
Ag thin film/Ti	LED (@365 nm, 170 mW cm^–2^)	1 M KHCO_3_	CO	–0.6 V_RHE_	0.62 mA cm^–2^ photo CD @ −1.1 V_RHE_	95%	459% for FE	(94)
Ag/Cu	LED (@365 nm, 170 mW cm^–2^)	0.1 M KHCO_3_	CO	–0.7 V_RHE_^a^	0.6 mA cm^–2^ partial CD^a^	68%^a^	79% for FE, 200% for CD	(270)
			ethylene	–1.0 V_RHE_^a^	0.9 mA cm^–2^ partial CD^a^	24%^a^	41% for FE, 13% for CD	
			CH_4_	–1.0 V_RHE_^a^	0.17 mA cm^–2^ partial CD^a^	4.5%^a^	36% for FE, 13% for CD	
			formate	–0.8 V_RHE_^a^	0.03 mA cm^–2^ partial CD^a^	3.1%^a^	15% for FE, 50% for CD	
			EtOH	–1.0 V_RHE_^a^	0.07 mA cm^–2^ partial CD^a^	1.7%^a^	13% for FE, 0% for CD	
			allyl alcohol	–1.0 V_RHE_^a^	0.007 mA cm^–2^ partial CD^a^	0.17%^a^	31% for FE	
AuNPs	LED (@520 nm, 120 mW cm^–2^)	0.5 M KHCO_3_	MeOH	–0.8 V_RHE_	3.75 mA cm^–2^ total CD^a^	52%	–, 49% for CD	(92)
Ag nanopyramids (180 nm)	Xe lamp (850 mW cm^–2^)	0.1 M NaClO_4_ (CO_2_-saturated)	CO, CH_4_	–1.1 V_RHE_	1.85 mA cm^–2^ total CD^a^	–	–, 9% for CD	(271)
Ag nanopyramids (75 nm)	Xe lamp (850 mW cm^–2^)	0.1 M NaClO_4_ (CO_2_-saturated)	MeOH, EtOH	–1.1 V_RHE_	1.05 mA cm^–2^ total CD^a^	–	–, 31% for CD	(271)

**PNP–Semiconductor for CO**_**2**_								
RA-Au/*n*^+^*p* Si	solar simulator (100 mW cm^–2^)	0.2 M KHCO_3_	CO	–0.59 V_RHE_	3.2 mA cm^–2^ total CD^a^	96%	–	(272)
Au/*p*^–^ GaN	visible light (λ > 495 nm, 600 mW cm^–2^)	50 mM K_2_CO_3_ (CO_2_-saturated)	CO	–1.8 V_RHE_	–	CO:H_2_ 5:1	CO:H_2_ 4:1 (dark), 5:1 (illumination)	(273)
Cu/*p*^–^ NiO	LED (λ = 565 ± 52 nm, 160 mW cm^–2^)	50 mM K_2_CO_3_ (CO_2_-saturated)	CO	–0.7 V_RHE_	0.18 mA cm^–2^ total CD^a^	19%^a^	217% for FE, 260% for CD	(62)
			formate	–0.7 V_RHE_	0.24 mA cm^–2^ partial CD^a^	22%^a^	267% for FE, 100% for CD	
Ag/Cu_2_O/CuO	LED (100 mW cm^–2^)	0.1 M Na_2_SO_4_ (CO_2_-saturated)	acetate	–0.4 V_Ag/AgCl_	0.55 mA cm^–2^ total CD^a^	54%	–, 244% for CD	(91)

a Values extracted from graph or calculated; LE is calculated as (value with illumination - value in dark)/value in dark.

The ability of plasmonic electrocatalytic systems to modify chemical reaction landscapes was also studied by Creel et al. on plasmonically active Ag thin film electrodes.^94^ They found that under illumination, the FE for CO increased to up to 95%, while in pure electrocatalysis it was only 17%. At the same time, the production of H_2_ was suppressed (from FE of 75% in dark to 10% under illumination), as shown in the left panel of Figure 7b.^94^ Moreover, methanol production was observed only under illumination (right panel of Figure 7b). The FE reached up to 2% (at −1.1 V_RHE_), which is reportedly an unprecedented value for silver cathodes. This shows that plasmonics provides a promising approach to promote complex electrochemical CO_2_RRs over other competing reactions.

Apart from the metallic electrodes discussed above, the application of metal–semiconductor hybrid materials in plasmon-assisted electrochemistry can potentially further enhance CO_2_ reduction. This was probed by Landaeta et al. with a plasmonic–metal semiconductor system, consisting of Ag and Cu_2_O/CuO (Ag/Cu_*x*_O).^91^ The illuminated hybrid system allowed the production of acetate with a FE of 54% at a potential of −0.4 V_Ag/AgCl_. The formation of acetate at such a low potential had not been reported before. Moreover, The current density reached 0.55 mA cm^–2^ upon illumination, which was 3.4 times as high as the value in dark. The enhanced performance of the hybrid originated from synergistic effects between the metal and the semiconductor—as no photoelectrocatalytic activity was observed on either just the Ag nor the Cu_*x*_O alone at such low potentials. The synergistic effect between Ag and Cu_*x*_O was attributed to reduced charge recombination processes thereby decreasing the needed overpotential. Additionally, Ag stabilized the Cu_2_O/CuO within the applied potential range, further improving the catalytic performance.

Cu is widely studied for electrochemical CO_2_ reduction because of its ability to catalyze the electrochemical conversion of CO_2_ to valuable fuels and chemicals beyond CO.^274^ To improve selectivity, Corson et al. coated Cu nanostructures with silver to create a plasmonically active cathode.^270^ Illumination allowed the selective enhancement of 5 of the 14 typically observed copper CO_2_ reduction products while simultaneously suppressing hydrogen evolution.^270^ Their Faradaic efficiencies can be found in Table 12. Under illumination, the CO production was enhanced at low overpotentials, whereas at high overpotentials the production of ethylene, methane, formate, and allyl alcohol was increased.

In conclusion, introducing plasmonics into electrocatalytic systems is a promising method to improve the activity of both HER and CO_2_RR and increase the selectivity of CO_2_RR. These properties allow the utilization of light to decrease the energy consumption in producing renewable and clean fuels. However, most plasmonic metals investigated in plasmon-assisted electrocatalytic systems are noble metals, which are costly and therefore hinder industrial applications. Additionally, in CO_2_RR the Faradaic efficiencies of hydrocarbons and multicarbon products are still low, which need to be improved in the future.

## Discussion

In this section, we will summarize and compare the performance of the hybrid plasmonic photocatalysts reviewed so far. We will organize the comparison according to the following aspects. First, we make a general review on the most frequently utilized plasmonic components and light sources in plasmonic catalysis. Second, we quantitatively compare photocatalysts according to their activity and selectivity, breaking down the different reactions of interest for hydrogen production and CO_2_ reduction. Third, we compare the performance of the different plasmonic electrocatalysts for HER and for CO_2_ reduction.

**Figure 8:**
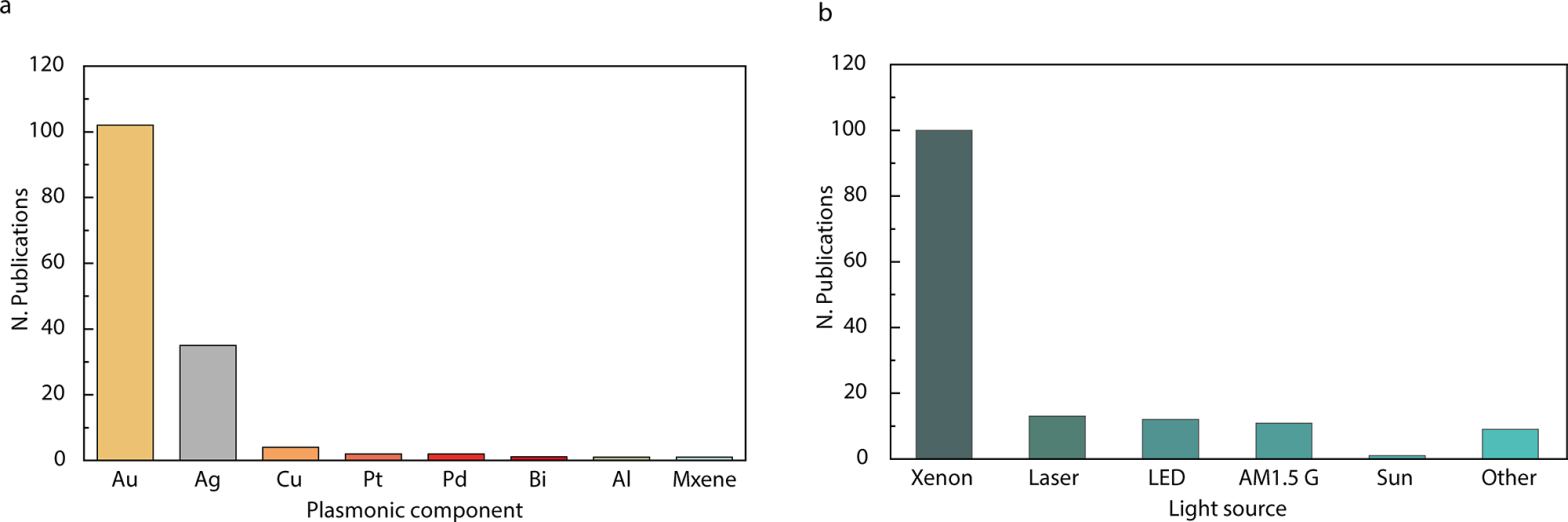
Histograms of (a) the main plasmonic component and (b) the illumination source used in the publications reviewed.

In the vast majority of the systems explored until now, the plasmonic component was either Au or Ag. Figure 8a shows a histogram of the main plasmonic component among the hybrid systems in this Review: 68% use Au, 24% use Ag, and only 8% use a different material. Reasons for this preference may include the high photostability of Au and the availability of versatile protocols to shape Au into different nanomorphologies with tailored resonances along the visible spectrum. Earth abundant plasmonic materials have been less explored.

Basing hybrid plasmonic catalysts on expensive, rare metals is a bottleneck in the development of sustainable large-scale photocatalytic methods. New abundant plasmonic materials like Al,^160,275,276^ Cu,^35,62,113,186,199^ Mg,^277^ metal oxides,^278−280^ metal nitrides^281^ or even 2D materials,^93^ are emerging candidates in plasmonic catalysis. However, they have been relatively unexplored until now. Moreover, hybrid combinations of these materials are a very interesting and emerging field to explore further. Research in these directions and with these materials could merge plasmonic catalysis with sustainable and large-scale solar-to-chemical energy conversion routes, disclosing the real potential for the industrial application of these concepts.

Another key parameter in (electro)photocatalysis is the light source. Figure 8b shows the histogram of illumination sources among the publications reviewed here. The most used is the xenon lamp, because its emission spectrum resembles the solar one in the wavelength range 290–700 nm. They account for 68% of the examples, followed by monochromatic laser light with 9%. Lasers are chosen to match the plasmonic absorption peak of the used photocatalyst and are a useful tool to study the wavelength dependence of their properties. In addition, they can provide higher irradiances. The third most frequent illumination source is light-emitting diodes (LEDs), 8%. 365 nm LEDs are particularly popular for electrochemical CO_2_ reduction, as they can induce the generation of allyl alcohol on Ag/Cu hybrids. Only 7% use AM 1.5 G sources, which are the standard for solar simulators.

Quantitative comparison among different photocatalysts is challenging due to the existent discrepancies in reported metrics and the high number of relevant experimental parameters involved, usually not reported.^104,105^ We believe that the field can benefit from standardization of reported activity metrics and measurement conditions. The adopted metric should incorporate information on the amount of catalyst and the intensity of the employed light. For this reason, we propose the implementation of the following activity metric:
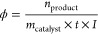
6where *n*_product_ is the amount of generated product, *t* is the time interval, *m*_catalyst_ is this mass of illuminated catalyst, and *I* is the incident light irradiance. This quantity will only be meaningful if measured far from saturation, i.e., with a linear dependence on time and catalyst concentration, in a large excess of reactants. Plasmonic systems have resonances, and therefore their activities are expected to be wavelength dependent. For this reason, the spectral composition of the illumination source must always be reported. Standardization of the employed light sources would allow a proper comparison among systems. The reasonable choice for the field of artificial photosynthesis would be AM 1.5 global light sources. Additionally, to investigate the role of the spectral composition of light on the efficiency, measuring single-wavelength yields using monochromatic irradiation may be useful.

It is important to compare plasmonic catalysts operating at similar bulk temperatures of the photoreactor. However, less than half (47%) of the publications reviewed here have reported a controlled reactor temperature. In addition, variations in the geometry of the photoreactors could also lead to discrepancies in the reported performances, even with the same employed catalyst.^282^ To overcome this issue, the field could benefit from the establishment of a standard photocatalyst, to be used as an internal reference at each laboratory, in a similar fashion as it was proposed for semiconducting photocatalytic materials.^283^

In many of the examples reviewed here, the information on reaction conditions is incomplete. In addition, even when reported, conditions vary among the different examples. Nevertheless, we believe that insightful conclusions can be obtained from the comparison of formation rates. Although the comparison between two photocatalysts is not exact under different conditions, we will focus here on evaluations of orders of magnitude in the produced formation rates. While doing so, we aim at identifying the best performers and the ones with the highest potential among the multiple hybrid plasmonic catalysts demonstrated in the past years.

**Figure 9:**
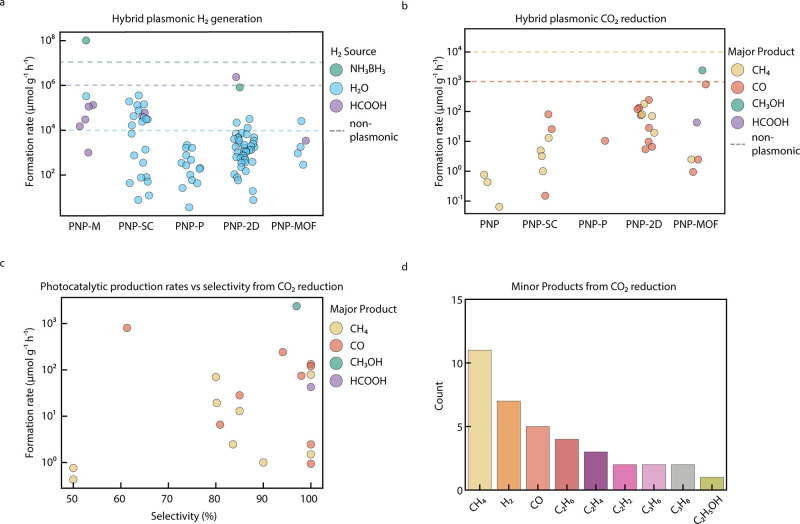
Hybrid plasmonic nanomaterials for the generation of solar fuels. Formation rates for (a) H_2_ generation and (b) CO_2_ reduction for the different hybrid photocatalysts reviewed here. The dashed line illustrates the order of magnitude achieved with prominent examples of non-plasmonic catalysts. (c) Formation rate vs selectivity and (d) histogram of the number of publications vs the minor products reported.

Figure 9 shows the formation rates extracted from all tables in this Review for photocatalytic H_2_ generation (Figure 9a) and CO_2_ reduction (Figure 9b). Exploration of plasmonic systems for H_2_ has been the most extensive, accounting for 71% of the publications reviewed here. Among the systems used for H_2_ production, the most popular were plasmonic metal–2D catalysts (38%), followed by metal–electrochemistry (25%), metal–semiconductor (18%), metal–perovskite (7%), metal–metal (7%), and metal–MOF (5%). For CO_2_ reduction, the most popular were also plasmonic metal–2D catalysts (25%), followed by metal–electrochemistry (20%), metal–semiconductor (18%), metal–metal (17%), metal–MOF (13%), and metal–perovskite (7%). The range of formation rates reported and the most efficient systems depend on the reaction under study. We will now break down the analysis into the different reactions of interest.

### Hybrid Plasmonic Nanomaterials for Photocatalytic Hydrogen Production

The performance of different plasmonic catalysts will be evaluated for the following three hydrogen production reactions: ammonia borane dehydrogenation, formic acid dehydrogenation, and water-splitting.

The hydrolytic dehydrogenation of ammonia borane was found to be the hydrogen generation reaction with the highest formation rate. An outstandingly high formation rate of 10^8^ μmol g^–1^ h^–1^ at 40 °C was observed employing core–shell Ag@Pd nanostructures.^109^ To put this number into context, one of the highest reported performances of heterogeneous catalytic systems for hydrolysis of ammonia borane at room temperature is 69.8 mol/min using porous Pt_25_Pd_75_NPs^284^ This translates to 3 × 10^7^ μmol g^–1^ h^–1^ if a molar ratio of 25% Pt and 75% Pd is assumed. Therefore, the core–shell Ag@Pd nanostructures offer a comparable performance with just a 1.3 nm layer of Pd and a relatively mild illumination source of 50 mW cm^–2^ (on the order of half the average solar irradiance).

The hydrogen generation from formic acid dehydrogenation was possible with formation rates of the order between 10^2^ and 10^6^ μmol g^–1^ h^–1^, as shown in Figure 9a. The metal–metal AgPd catalyst loaded on 2D graphitic carbon nitride nanosheets achieved the highest formation rate (2 × 10^6^ μmol g^–1^ h^–1^).^187^ Furthermore, AuNPs interfaced with a metal (Pd) or semiconductor (CdS) were also capable of photocatalyzing formic acid dehydrogenation with formation rates in the order of 10^5^ μmol g^–1^ h^–1^.^114,132^ However, metal–semiconductor systems were able to reach these formation rates at room temperatures, while the highest activities in metal–metal systems required temperatures between 40 and 50 °C. In addition, semiconductors are typically cheaper and more abundant than catalytic metals, indicating that metal–semiconductors systems could be preferable for this reaction over metal–metal systems. Overall, the best activities for photocatalytic hydrogen generation from formic acid with plasmonic systems are comparable to the activity of heterogeneous catalysis at room temperature and without light, where formation rates on the order of 10^6^ μmol g^–1^ h^–1^ were also achieved, e.g., using 2.2 nm Ag_42_Pd_58_ NPs.^285^

Reviewing H_2_ generation from water-splitting reactions, we observed formation rates of the order between 10^0^ and 10^5^ μmol g^–1^ h^–1^. The highest activity was 3.5 × 10^5^ μmol g^–1^ h^–1^, achieved using plasmonic PtNPs incorporated in nanostructured TiO_2_,^130^ closely followed by a dual metal–semiconductor system including both Au@CdS and Ag@SiO_2_ and achieving 2 × 10^5^ μmol g^–1^ h^–1^ at a temperature of 15 °C.^132^ In addition, using metal–metal ZnCu plasmonic alloys, formation rates on the order of 10^5^ μmol g^–1^ h^–1^ were also achieved, illustrating an interesting demonstration that does not utilize Au or Ag.^113^ . Another example that does not use Au or Ag as the plasmonic component are CuNPs interfaced with WS_2_ nanosheets that produced 10^4^ μmol g^–1^ h^–1^ of hydrogen under 1 solar irradiation.^186^ Finally, it has been shown that it is possible to drive water-splitting reactions using perovskites. However, up to date, most examples still show limited efficiencies in the order of 10^3^ μmol g^–1^ h^–1^. Remarkably, the highest plasmonic activities for water-splitting are 1 order of magnitude larger than the best activities achieved by other non-plasmonic graphitic carbon nitride-based photocatalysts.^286,287^ This demonstrates that plasmonic nanomaterials have a substantial potential for water-splitting reactions.

### Hybrid Plasmonic Nanomaterials for Photocatalytic CO_2_ Reduction

As shown in Figure 9b, the formation rates for CO_2_ reduction are in general lower than for hydrogen production, in the range of 10^–1^–10^3^ μmol g^–1^ h^–1^. Moreover, we can observe that the main product of many catalysts for CO_2_ reduction is CO (56%), followed by CH_4_ (32%). Figure 9c shows formation rate of each reactions main product versus its selectivity. The plot has less points than Figure 9b because selectivity is less commonly reported. Interestingly, in the cases where it is reported, it is higher than 50%. It must be noted that there is a lack of examples where products with more than one carbon (such as C_2_H_6_, C_2_H_4_, and C_3_H_6_) are produced with high selectivity. However, these chemicals have been obtained as minor products in numerous publications. Figure 9d shows the reported minor products for CO_2_ reduction, showing that C–C coupling reactions are indeed possible to achieve with plasmonic catalysts. This is a very interesting but poorly explored route so far.

To date, the highest reported performance for producing CO was obtained using AgNPs deposited onto a MOF. Specifically, MIL–101(Cr)/Ag showed a CO production of 8 × 10^2^ μmol g^–1^ h^–1^ at 50 °C, with a selectivity of 61.3%.^241^ The hybrids with the next best production rates are plasmonic–2D Ag/rGO achieving 10^2^ μmol g^–1^ h^–1^ at 45 °C, with a high selectivity close to 100%.^184^ Furthermore, plasmonic-semiconductor systems formed by Au/CdSe-Cu_2_O obtained a CO formation rate of 80.2 μmol g^–1^ h^–1^.^123^ It should be noted that to date, there have been no reports of bimetallic PNPs for this reaction. The highest activity obtained with plasmonic hybrids is comparable to what can be achieved by a non-plasmonic g-C_3_N_4_ nanosheet with B–H bonding decorated MOF (∼10^3^ μmol g^–1^ h^–1^).^288^

The second main product obtained from the reduction of CO_2_ was CH_4_. This reaction requires multiple electron transfers, and therefore CH_4_ is more difficult to produce than CO. The best catalysts for producing CH_4_ were metal–2D materials. All examples exhibited formation rates on the order of 10^2^. A system consisting of AgNPs combined with N-doped TiO_2_ nanosheets achieved a formation rate of 79 μmol g^–1^ h^–1^ with almost 100% selectivity. Another plasmonic-2D material Au/MoS_2_, also showed CH_4_ production rate on the same order of magnitude, achieving 20 μmol g^–1^ h^–1^ with a selectivity of 98%. From the metal-semiconductor category there was one example achieving that order of magnitude with 13 μmol g^–1^ h^–1^. It must be noted that CH_4_ production from CO_2_ was also demonstrated with metal and metal–MOF systems but with lower activities. The formation rates for CH_4_ production with plasmonic systems are still lower than the ones recently achieved by non-plasmonic photocatalytic systems such as g-C_3_N_4_ nanosheet with B–H bonding decorated MOF (1.5 × 10^4^ μmol g^–1^ h^–1^) or titanium carbide compounds (786 μmol g^–1^ h^–1^).^288,289^

Finally, there are two examples worth mentioning where methanol and formic acid were obtained as main products with close to 100% selectivity using plasmonic-MOF systems. First, AuNPs incorporated in a zeolitic imidazolate framework (ZIF-67) produced 2 × 10^3^ μmol g^–1^ h^–1^ of methanol under irradiation from a solar simulator.^87^ Second, hybrids of porphyrin frameworks loaded with AuNPs produced 40 μmol g^–1^ h^–1^ of formic acid at room temperature.^240^ These two examples show the great potential of plasmonic–MOF systems for selective reduction of CO_2_ into solar fuels.

### Hybrid Plasmonic Nanomaterials for Photoelectrocatalytic H_2_O Splitting and CO_2_ Reduction

**Figure 10:**
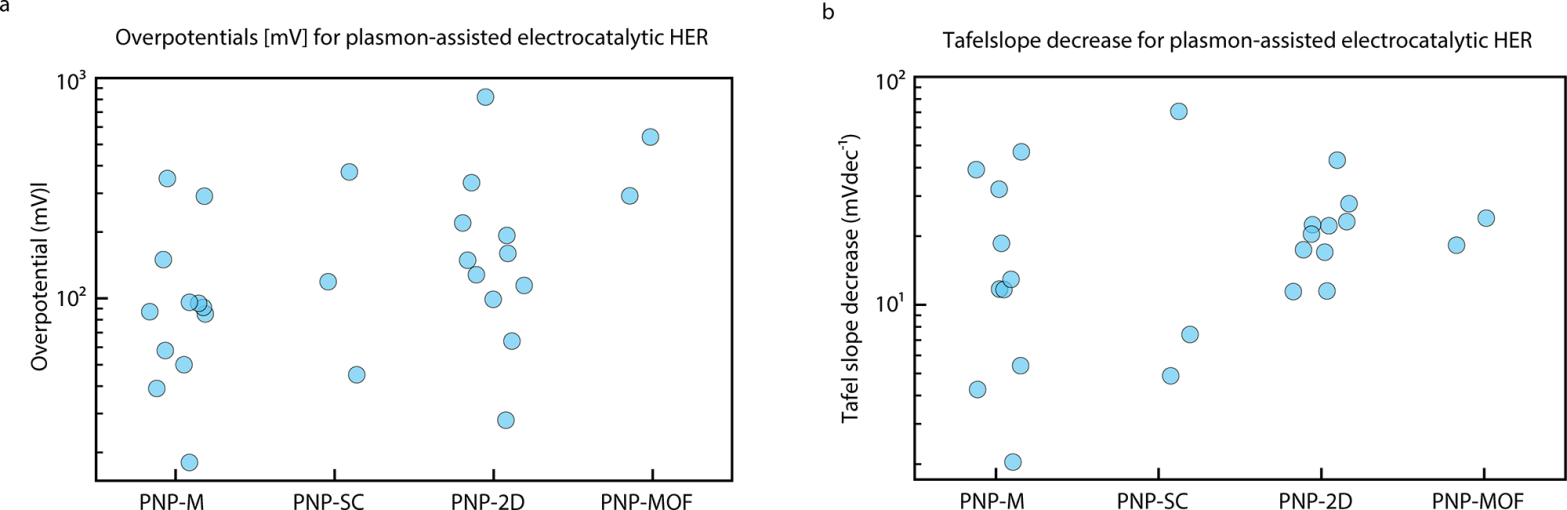
(a) Overpotential for plasmon-assisted electrocatalytic HER for all hybrid systems reviewed here. (b) Tafel slope decrease upon illumination of hybrid plasmonic electrocatalysts.

Illuminated plasmonic nanomaterials can successfully decrease the overpotentials required for electrocatalytic hydrogen evolution. Figure 10a summarizes the required overpotentials of all the plasmonic system reviewed here, at a current density of 10 mA cm^–2^. Notably, there are several examples of metal–metal hybrids requiring overpotentials lower than 100 mV. The best performer plasmonic system was metal–metal Pt/Fe@Au nanorods that were able to reduce the overpotential to 18 mV under irradiation.^215^ In addition, this system showed a Tafel slope of 29.3 mV/dec, which is close to the theoretical minimum of the Tafel slope in hydrogen evolution from aqueous solution.^290^ Plasmonic metals in combination with semiconductors and 2D materials were also capable of delivering low overpotentials. Upon illumination, the overpotential was 45 mV for nanoperforated Au films on a Ti/TiO_2_ film, due to the facilitated electron transfer at the Au and TiO_2_ Schottky barrier.^257^ By employing Au nanorods on a graphene/MoS_2_ substrate it was possible to lower the required overpotential to 28 mV.^259^ On the other hand, MOFs systems presented larger overpotentials above 292 mV. Although MOFs with large specific surface area could provide more catalytic sites for plasmonic electrocatalysts, their usually high resistivity retards the hydrogen evolution activity.

One way to evaluate the role of light in photoelectrocatalysis is to measure the Tafel slope decrease under illumination. Figure 10b shows the Tafel slope reduction of every system in this Review. Decreases are most significant in metal–2D hybrids, highlighting the beneficial contribution of plasmonics to promote photoexcited charge separation in these materials.

Overall, it can be concluded that metal–metal and metal–2D hybrid nanostructures have shown the greatest photoelectrocatalytic performances and are the most promising materials for catalyzing the HER.

Plasmonic systems are also beneficial to enhance CO_2_ electroreduction. However, up to date, we were able to find only eight published examples. Among these, some remarkable performances can be highlighted. For example, by using a metal–metal Ag/Cu hybrid plasmonic system, it was possible to enhance the CO partial current density by 200% when illuminated.^270^ In another example, using a Cu/p-NiO metal–semiconductor hybrid, the partial current density values of CO and HCOOH increased by 260% and 100% under illumination.^62^

Regarding the selectivity of CO_2_ reduction, most reviewed plasmonic electrocatalysts displayed an improved FE toward specific products. For example, the FE of carbon monoxide and formate on Cu/p-NiO with illumination increased by 217% and 267%, respectively.^62^ Another interesting example of improved FE was the achieved 95% CO FE of Ag/Ti film under 365 nm LED illumination at −0.6 V_RHE_.^94^ This performance is better than that of one of the top pure electrocatalysts: nanoporous Ag achieved 92% CO FE at −0.6 V.^291^ These results indicate that metal–semiconductor hybrids are promising systems to improve CO_2_ electroreduction activity and selectivity by plasmon excitation.

In spite of the advances made in the design of hybrid plasmonic nanomaterials for photoelectrocatalytic CO_2_ reduction and hydrogen evolution, the role of the plasmon is still unclear. In pure electrocatalytic CO_2_ reduction, the interface between metal and oxide semiconductor is usually considered as the active catalytic center as it facilitates the stabilization of reaction intermediates.^292^ Plasmon excitation is expected to be beneficial for CO_2_ reduction at the interface, because the photothermal effect and electromagnetic field enhancement could facilitate the CO_2_ activation by lowering the reaction energy barrier.^293^ However, the exact role of plasmon excitation on the stabilization of CO_2_ intermediates is still elusive. More detailed investigation should be focused on unraveling the origin of the plasmonic enhancement. For example, electrochemically in situ Raman^190^ and Fourier transform infrared spectroscopy (FTIR)^270^ can be employed to unveil the plasmon-enhanced photoelectrocatalytic reaction mechanisms. These methods employed with and without light irradiation could provide some insight into how the plasmon excitation changes the reaction pathway of electrocatalytic processes on plasmonic electrodes. Additionally, a significant enhancement of photocurrent is crucial for the improvement of plasmon-assisted photoelectrocatalytic performance. However, the short lifetime of hot carriers in plasmonic nanomaterials results in a low incident photon-to-electron conversion efficiency (IPCE), which limits the activity enhancement in plasmon-assisted photoelectrocatalytic hydrogen evolution and CO_2_ reduction. To this end, the interface between components in hybrid plasmonic nanostructures that can enhance the hot electron–hole separation efficiency is a key factor to achieve a high plasmonic enhancement.^13,115,117^ Therefore, it is pivotal to optimize the interface between plasmonic metals and hybrid components to prolong the lifetime of hot electrons generated from the plasmon dephasing. Consequently, more efforts should be made to explore the materials with high electron mobility or long electron diffusion length as the candidate to couple with plasmonic metals.

### Outlook

We have critically reviewed and benchmarked a large set of recently reported hybrid plasmonic materials for solar fuel production. Remarkably, the hydrogen formation rates achieved by plasmonic catalysts are comparable to state of the art technologies, with the advantage of generally employing milder reaction conditions or less expensive materials, such as metal–semiconductor or metal–2D hybrids. However, most of these examples were performed with irradiances larger than expected for sunlight-driven catalysis. To reach or surpass these formation values with irradiances comparable to sunlight, further development of sunlight concentrators or focusing devices is still needed as well as novel reactor designs that can maximize light penetration and absorption. Scalability of these technologies seems possible but is scarcely explored so far. On the other hand, carbon dioxide reduction with plasmonic catalysts has not yet shown such appealing results as hydrogen generation. In general, the selectivity and efficiency for this reaction when employing hybrid plasmonic materials are still far from those of the excellent electrocatalysts reported in the literature. In this sense, the combination of plasmonic materials with metal organic frameworks appears as a promising route to explore, with few but remarkable results so far. Additionally, the combination of more than two types of materials into higher order plasmonic hybrid systems may not only allow the exploitation of the properties of the respective two-component hybrid systems but also introduce new synergies.^85,180^ Finally, even though we have only focused on solar fuels, plasmonic hybrids might present interesting opportunities for the production of highly valuable chemicals that in most cases require the use of expensive precious metal (electro)catalysts.^294^ In this context, earth-abundant plasmonic materials have been rarely explored for catalysis; Al, TiN, some metal oxides, among others are materials that deserve further attention. Interestingly, plasmonic materials as support of single catalytic atoms could also become a promising route for the future development of the field.^295^ From the analysis of the systems reported here, the combination of plasmonic materials with also earth-abundant hybrid materials, such as nitrides (TiN, g-C_3_N_4_) and MOFs, may lead to new routes toward efficient and selective sustainable catalysis.

## Notes

Author group website: https://www.hybridplasmonics.org/.
